# Host-dependent nitrogen recycling as a mechanism of symbiont control in *Aiptasia*

**DOI:** 10.1371/journal.pgen.1008189

**Published:** 2019-06-24

**Authors:** Guoxin Cui, Yi Jin Liew, Yong Li, Najeh Kharbatia, Noura I. Zahran, Abdul-Hamid Emwas, Victor M. Eguiluz, Manuel Aranda

**Affiliations:** 1 King Abdullah University of Science and Technology (KAUST), Red Sea Research Center (RSRC), Biological and Environmental Science & Engineering Division (BESE), Thuwal, Saudi Arabia; 2 King Abdullah University of Science and Technology (KAUST), Core Labs, Thuwal, Saudi Arabia; 3 Instituto de Física Interdisciplinar y Sistemas Complejos IFISC (CSIC-UIB), Palma de Mallorca, Spain; Eckerd College, UNITED STATES

## Abstract

The metabolic symbiosis with photosynthetic algae allows corals to thrive in the oligotrophic environments of tropical seas. Different aspects of this relationship have been investigated using the emerging model organism *Aiptasia*. However, many fundamental questions, such as the nature of the symbiotic relationship and the interactions of nutrients between the partners remain highly debated. Using a meta-analysis approach, we identified a core set of 731 high-confidence symbiosis-associated genes that revealed host-dependent recycling of waste ammonium and amino acid synthesis as central processes in this relationship. Subsequent validation via metabolomic analyses confirmed that symbiont-derived carbon enables host recycling of ammonium into nonessential amino acids. We propose that this provides a regulatory mechanism to control symbiont growth through a carbon-dependent negative feedback of nitrogen availability to the symbiont. The dependence of this mechanism on symbiont-derived carbon highlights the susceptibility of this symbiosis to changes in carbon translocation, as imposed by environmental stress.

## Author summary

The symbiotic relationship with photosynthetic algae is key to the success of reef building corals in the nutrient poor environment of tropical waters. Extensive insight has been obtained from both physiological and “omics” level studies, yet, there are still gaps in our knowledge with respect to the metabolic interactions in this symbiotic relationship. In particular, the role of the host in nitrogen utilization and its potential link to symbiont population control still remains unclear. Using a meta-analysis approach on publicly available RNA-seq data and isotope-labeled metabolomics, we demonstrate the presence of a negative-feedback cycle in which the host uses symbiont-derived organic carbon to assimilate its own waste ammonium. This host-driven nitrogen recycling process might serve as a molecular mechanism to control symbiont densities *in hospite*. The dependence of this regulatory mechanism on organic carbon provided by the symbionts explains the sensitivity of this symbiotic relationship to environmental stress.

## Introduction

The symbiotic relationship between photosynthetic dinoflagellates in the family Symbiodiniaceae [1] and corals is the foundation of the coral reef ecosystem. This metabolic symbiosis is thought to enable corals to thrive in the oligotrophic environment of tropical oceans by allowing efficient recycling of nitrogenous waste products in return for photosynthates from the symbionts [2]. Despite the general acceptance of this assumption, cumulative studies have raised discussions about the molecular mechanisms underlying host-symbiont metabolic interactions.

In particular, the role of nitrogen recycling from waste ammonium is still under debate. While it is generally assumed that ammonium assimilation is predominantly performed by the symbiont, some studies indicate that symbiont-growth is nitrogen limited *in hospite* [3–5], suggesting that the host might be able to control nitrogen availability. Moreover, it has been suggested that the host might be able to utilize organic carbon [5], in the form of glucose provided by the symbiont [6], to promote ammonium assimilation by itself, while suppressing ammonium production from deamination reactions [7]. Consequently, it has been proposed that recycling of ammonium waste by the host might serve as a mechanism to control symbiont densities [5]. Although this potential mechanism to control symbiont densities through nitrogen conservation by the host has been proposed for decades, it still remains highly contentious. Consequently, the coral research field still recognizes nitrogen recycling as a main function of the symbiont [8, 9].

To better understand these metabolomic interactions, the sea anemone *Aiptasia* (sensu *Exaiptasia pallida*) [10]—an anthozoan as corals—has emerged as a powerful model system because of the similar symbiotic relationship it forms with Symbiodiniaceae. Multiple symbiosis-centered transcriptomic studies have provided invaluable information on the interactions between *Aiptasia* and Symbiodiniaceae [11–13]. To generate a more concise set of high-confidence symbiosis-related genes, we adapted a meta-analysis approach, which is a statistical method developed from evidence-based medical research [14]. Because of its statistical power in integrating results from multiple sources, it has been recently applied to transcriptomic studies from both animals [15] and plants [16], and allows for the identification of high-confidence genes associated with certain biological processes.

By carrying out a meta-analysis on available symbiosis-centered RNA-seq datasets, we identified a core set of high-confidence genes and pathways involved in symbiosis establishment and maintenance. To further verify our conclusions made from expression changes of these core genes, we subsequently analyzed metabolomic profiles of symbiotic and non-symbiotic *Aiptasia* using ^13^C bicarbonate labeling. Through the integration of these two layers of omics-level information, we identified the pathways associated with nitrogen conservation in the host animal, and further revealed competition for nitrogen as a central mechanism in this relationship that is generally believed to be entirely mutualistic. Based on these findings, we propose a glucose-dependent nitrogen competition model that highlights the sensitivity of the symbiotic relationship to environmental stresses.

## Results

To carry out the meta-analysis, we collected symbiosis-centered transcriptomic data that was generated from the same clonal *Aiptasia* strain CC7. Since this was the strain used to sequence the genome, we expected to minimize background noise in our meta-analysis by mapping the reads from the different transcriptomic studies to the published reference gene models [12]. Based on these requirements, we identified 3 previous RNA-seq studies that met our criteria and provided 4 separate datasets, encompassing 17 biological replicates per symbiosis state (i.e., aposymbiotic and symbiotic) [11–13]. We named the four datasets after the initials of the respective first authors whose paper we obtained the data from—YL, SB, EML, and EML-36 (i.e. Yong Li [13], Sebastian Baumgarten [12], and Erik M. Lehnert [11], respectively). The meta-analysis was conducted on the expression levels of gene models that were quantified based on these data.

### Experimental variations

**Fig 1:**
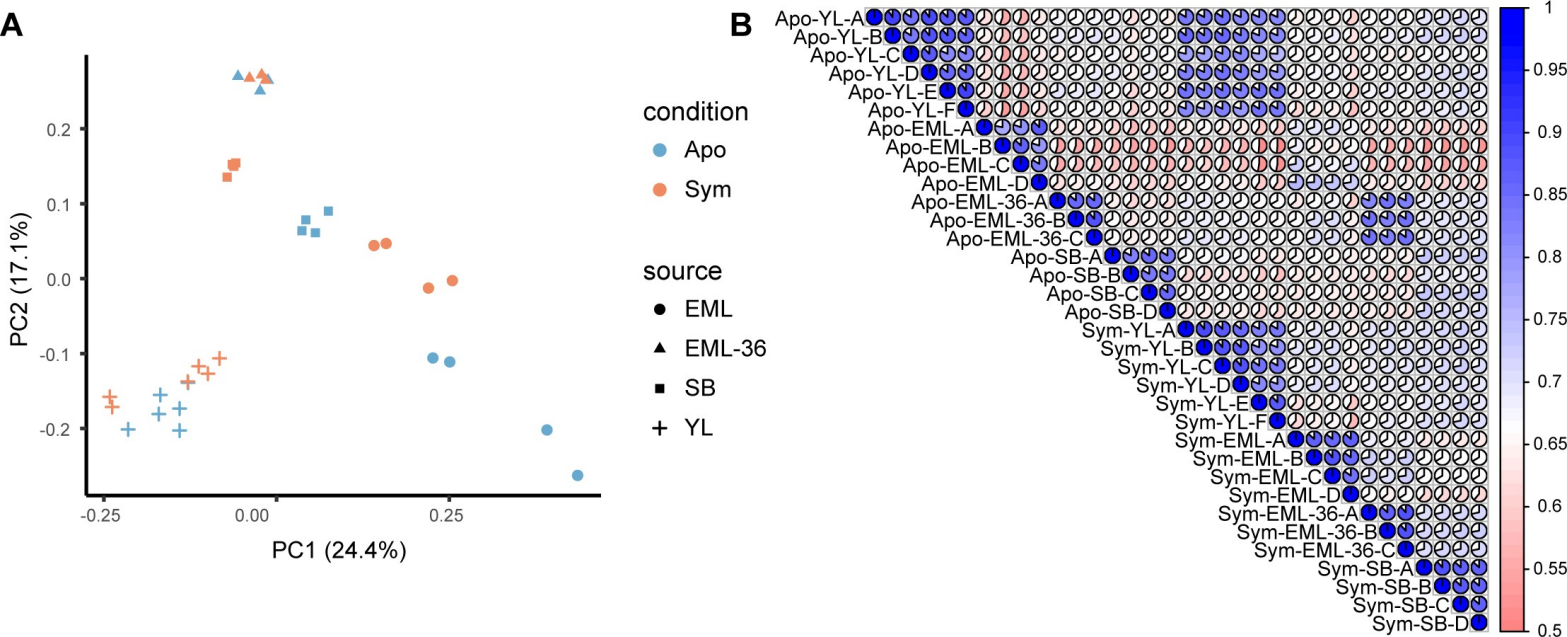
Relationship between samples from different studies. (A) Principal component analysis of samples across all four studies. The symbiotic state (condition) of the animals was indicated by the color of the points, while the source studies were represented as different shapes. (B) Kendall rank correlation of all samples, with high-correlation as blue, and low-correlation as red. The pie chart in each cell also indicates the correlation of the two samples from the corresponding row and column. In both figures, Apo and Sym represent the symbiotic state of the anemones: aposymbiotic and symbiotic, respectively.

The initial PCA performed on samples from individual studies showed a clear separation of samples by symbiotic condition (S1 Fig**)**. This implied that the symbiotic state was the main driver of expression changes in each of the individual studies. To further investigate the relationship between samples from different studies, we performed a principal component analysis (PCA) and a rank correlation analysis (RCA) on inter-sample normalized transcripts per million (TPM) values across all studies. Both the PCA (Fig 1A) and RCA (Fig 1B) showed clear grouping of samples by experiment rather than symbiotic state. This indicated that technical and/or experimental effects from each study exerted stronger effects on gene expression profiles than the actual symbiotic state of the animals.

### Differential expression analyses

Although the four datasets grouped distinctly in the PCA analysis (Fig 1), there was still a clear separation of symbiotic and aposymbiotic replicates within each of the datasets (S1 Fig). We hypothesized that this separation was due to the differential expression of core genes involved in symbiosis initiation and/or maintenance. To identify these genes, we performed four independent differential expression analyses using the exact same pipeline and parameters as described in Materials and Methods. These analyses identified between 2,398 to 11,959 differentially expressed genes (DEGs), corresponding to ~10–50% of all expressed genes in the respective studies (Table 1). Since the symbiotic state was supposed to be the main factor driving expression differences between the individuals in each study, we expected to find a great overlap between these lists of DEGs. However, the overlap was poor despite the large number of DEGs identified in the individual analyses: only 393 genes were consistently differentially expressed across all four studies. Out of these 393 genes, 166 were upregulated in symbiotic anemones in all comparisons, while 134 were found to be downregulated in symbiotic animals, relative to aposymbiotic controls (Table 1). Paradoxically, the remaining 93 genes (23.7% of all overlapped DEGs) were differentially expressed in all studies, but in different directions i.e. in some studies they were significantly upregulated while in others they were significantly downregulated.

**Table 1:** Number of differentially expressed genes in different analyses. “Upregulated” and “downregulated” refers to the number of genes that are expressed at higher levels and lower levels respectively in symbiotic *Aiptasia*, relative to aposymbiotic ones.

Study	Expressed	DEGs	Upregulated	Downregulated
**YL**	27,684	3,058	1,552	1,506
**SB**	24,013	11,959	6,072	5,887
**EML**	24,511	9,613	4,758	4,855
**EML-36**	24,246	2,398	1,241	1,157
**Overlap**	22,394	393	166	134
**Meta-analysis**	25,857	731	366	365

### Performing a meta-analysis across four datasets

To obtain a more robust set of core genes involved in symbiosis, we performed a meta-analysis with random effects across the four independent differential gene expression analyses (S1 Table). Using this approach, we identified 731 genes that exhibited a more consistent response to symbiosis.

**Fig 2:**
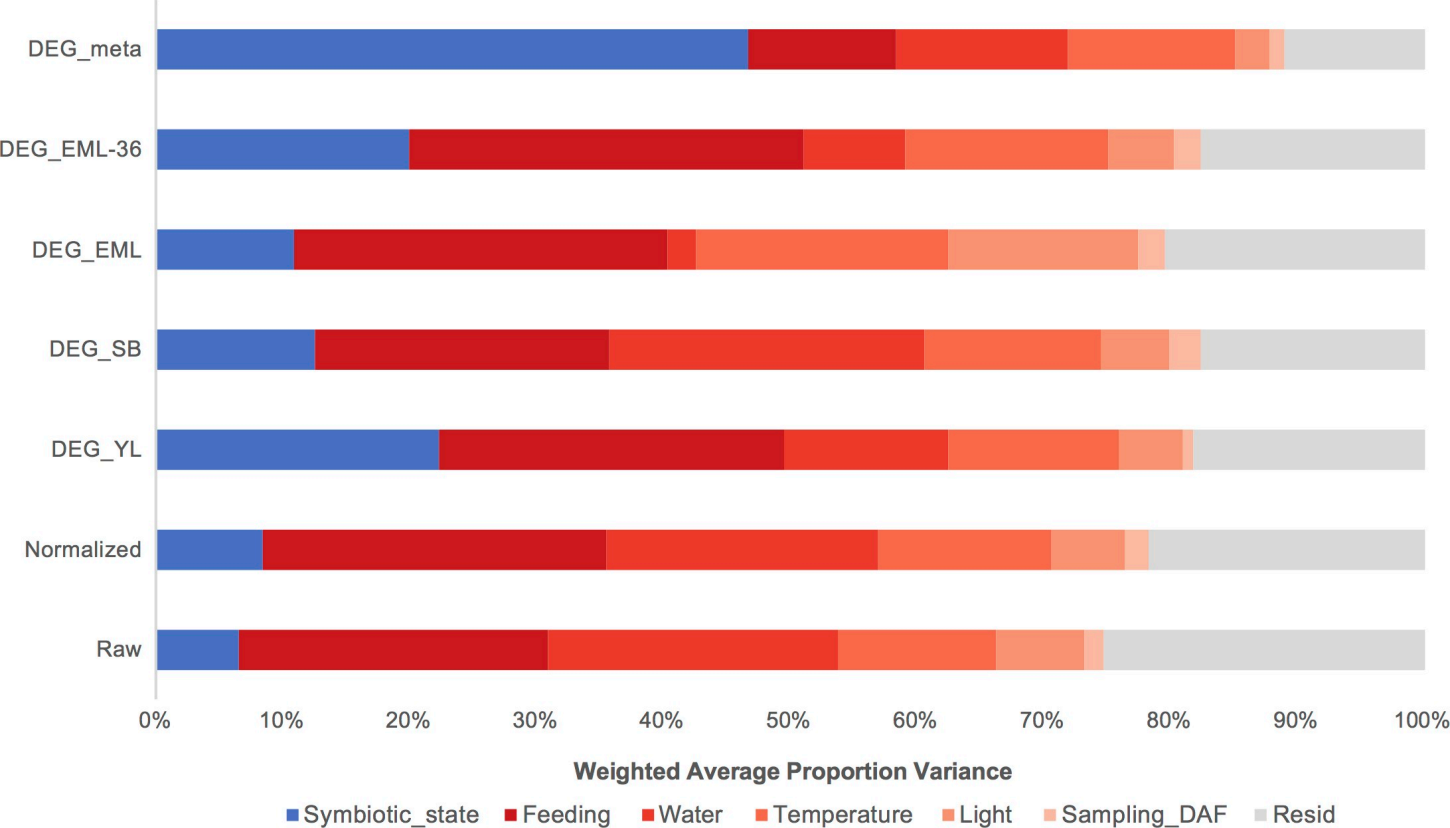
Principal variance component analysis of DEGs from different analyses. The contribution of each factor to the overall variance in each analysis was estimated by PVCA. The variance explained by symbiotic state (blue) is highest in the set of DEGs from the meta-analysis (DEG_meta); the combined variation attributable to experimental factors (red) is lowest in DEG_meta as well. Unresolved variance is in gray. DEG_YL, _SB, _EML and _EML-36 represent the sets of differentially expressed genes identified in four independent differential analyses. Raw and Normalized are the combined raw and inter-sample normalized expression data across all *Aiptasia* genes, showing that < 10% of the variation in overall gene expression can be attributed to symbiotic state. DAF: days after feeding.

To assess the robustness of these genes, we carried out a principal variance component analysis (PVCA). PVCA is an approach to partition the total variance present in the expression data by estimating the contribution of each experimental parameter (biological or technical) to the variance, and determine which of these sources are the most prominent [17]. By fitting the expression profiles and the different experimental parameters used in each study (such as feeding schedule, water source, temperature, etc., as shown in S2 Table) into the PVCA, we were able to detect correlations between expression changes and potential effect sources (Fig 2).

For the four individual studies, we found that the symbiotic state of the anemones accounted for a relatively small fraction (6.5% in raw data, 8.4% in normalized data) of the observed variance. Most of the variance was introduced by study-specific variables such as feeding frequency, days between feeding and sampling, water, light intensity, and temperature. We further noticed that a large proportion of the variance across these four datasets remained unaccountable, suggesting that technical variability, e.g. RNA extraction, library preparation and sequencing, also introduces substantial unwanted heterogeneity to gene expression profiles. When the PVCA was similarly applied to the 731 genes identified through our meta-analysis, we observed that these core genes had a significantly higher association with symbiosis. Symbiosis state accounted for 46.6% of the expression variance observed in these genes (Fig 2).

We noticed that smaller gene lists tended to have variances that were better explained by symbiosis state, exemplified by DEG_YL and DEG_EML-36 having better association with symbiosis than DEG_SB and DEG_EML (Fig 2). Thus, one could argue that the meta-analysis merely achieved better association with symbiosis as it had the fewest genes of interest. To assess this potentially confounding factor, we performed PVCAs on sets of 731 randomly picked genes from each of the DEG lists (DEG_YL, DEG_SB, DEG_EML, and DEG_EML-36). These were repeated 10,000 times (i.e., a Monte-Carlo approach). These simulations allowed us to estimate that the likelihood of our meta-analysis producing the observed 46.6% by random chance was *p* < 10^−4^ (0 of 40,000 trials had symbiosis state accounting for > 46.6% of the variance).

### Functional interpretation

To assess the impact of the previously identified experiment-specific biases, we conducted Gene Ontology (GO) and Kyoto Encyclopedia of Genes and Genomes (KEGG) enrichment analyses on the DEGs identified using the four independent differential gene expression analyses, respectively. Across the analyses of four independent experiments, 283–645 GO terms and 9–55 KEGG pathways were enriched. However, the functional overlap across all studies was poor: a large proportion of the putatively enriched terms were only identified in a single dataset (~75% in GO, and ~65% in KEGG) (S2 Fig). This finding reflected the previously observed poor overlap of differentially expressed genes across the studies and provided further evidence for the role of study-specific technical factors in driving gene expression profiles. Compared to the independent analyses, the GO and KEGG enrichment of the 731 symbiosis-associated core genes contained fewer significant GO terms (204) (S3 Table), but comparatively more significantly enriched KEGG reference pathways (31) (S4 Table).

Many of the enriched GO terms and KEGG reference pathways, as well as their associated genes, fit well with processes previously reported to be involved in symbiosis [11, 18], including symbiont recognition and the establishment of symbiosis, host tolerance of symbiont, and nutrient exchange between partners and host metabolism which are discussed separately (S1 Text). The enrichment of the KEGG nitrogen metabolism reference pathway (S3 Fig) concurs with previous studies that reported the symbiosis-induced upregulation of genes involved in glutamine synthetase / glutamine oxoglutarate aminotransferase (GS/GOGAT) cycle in *Aiptasia* [11, 18]. The GS/GOGAT cycle has been demonstrated to be the main pathway of ammonium assimilation in plants [19], bacteria [20] as well as in cnidarians [9, 11]. Moreover, we found that the GS/GOGAT cycle connects to several symbiosis-related processes that were previously overlooked; of these processes, pathways associated with amino acid metabolism exhibited some of the most extensive changes in response to symbiosis. These findings therefore suggested that amino acid biosynthesis pathways might play a previously undiscovered role in the maintenance of the symbiotic relationship.

### Extensive changes of amino acid metabolism in response to symbiosis

**Fig 3:**
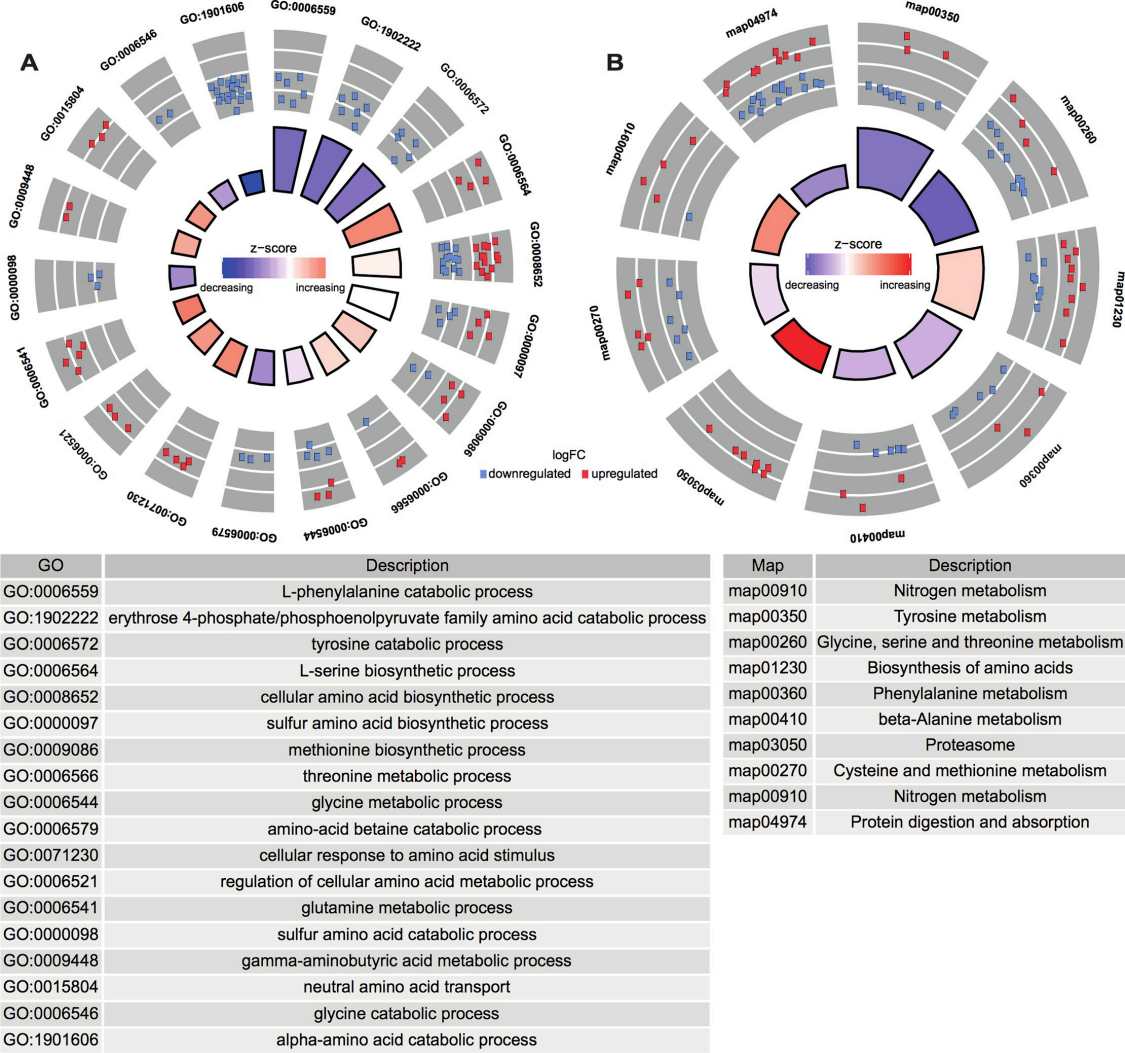
**Amino acid metabolism biological processes (A) and pathways (B) enriched with DEGs identified in meta-analysis.** For the two Circos plots, the height of each bar in the inner circle indicates statistical significance of the enriched GO terms (A) and KEGG pathways (B), while color of the bars represents the overall regulation effect of each process. The outer circle shows the differential expression of genes associated with each process, where red and blue represent upregulation and downregulation in symbiotic anemones, respectively. The table describes the annotation of each term or pathway.

Amino acid and protein metabolism represented a major symbiosis-related aspect in our meta-analysis. Nine of 31 enriched KEGG pathways (S4 Table) and 18 of 125 enriched biological process GO terms (S3 Table) were associated with amino acid and/or protein metabolism (Fig 3). A total of 97 DEGs were involved in these processes, of which 43 were upregulated in symbiotic animals. Interestingly, the DEGs involved in most of the enriched biological processes exhibited consistent expression changes (Fig 3A), i.e. the genes associated with the corresponding process were either exclusively upregulated or downregulated.

**Fig 4:**
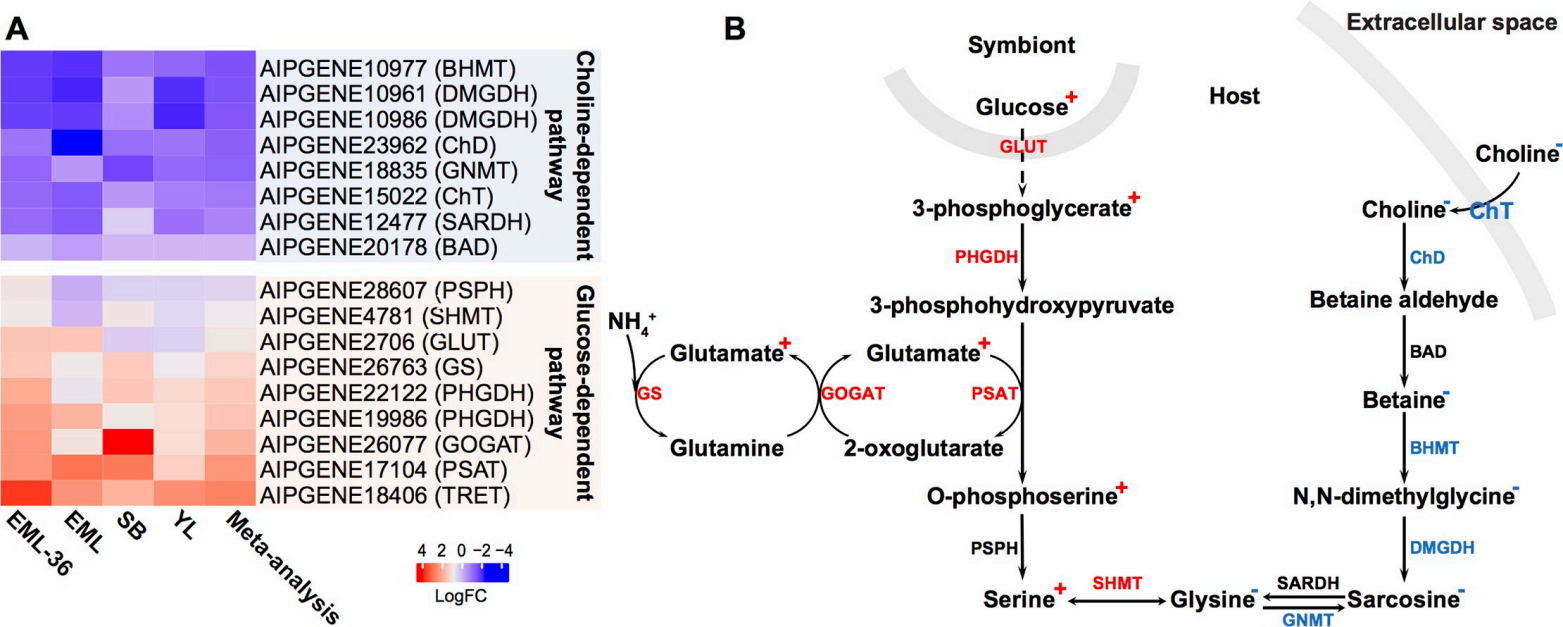
Amino acid biosynthetic pathways in response to *Aiptasia*-Symbiodiniaceae symbiosis. (A) Differential expression of genes involved in two alternative serine/glycine synthesis pathways: glucose-dependent and choline-dependent. Color indicates log-transformed fold change of the gene expression level in the comparison between symbiotic and aposymbiotic anemones. (B) Serine biosynthesis in symbiotic and aposymbiotic *Aiptasia*. The pathway on the left shows *de novo* serine biosynthesis using symbiont-produced glucose and waste ammonium, while the right part represents glycine/serine biosynthesis using food-derived choline. Enzyme names are colored to indicate differential expression of the corresponding genes, where red and blue mean upregulation and downregulation in symbiotic anemones, respectively. The changes in abundances of metabolites in symbiotic animals are indicated with +/- superscripts.

Further integration of these enriched biological processes and pathways revealed an amino acid metabolism hub in *Aiptasia*-Symbiodiniaceae symbiosis (Fig 4). We observed that genes catalyzing glycine/serine biosynthesis from food-derived choline were systematically downregulated in symbiotic anemones. In contrast, the genes involved in *de novo* serine biosynthesis from 3-phosphoglycerate, one of the glycolysis intermediates, and glutamine/glutamate metabolism were generally upregulated (Fig 4A). The resulting change in amino acid synthesis pathways suggested that symbiotic anemones utilize glucose and waste ammonium to synthesize serine and glycine, which are both main precursors for many other amino acids (S1 Text). Based on these findings, we hypothesized that the host might be using symbiont-derived glucose to assimilate waste ammonium into amino acids. To test this hypothesis, we further analyzed the metabolic profiles of anemones at different symbiotic states using ^13^C bicarbonate labeling, which can only be fixed by the symbiont through photosynthesis.

### Abundance and isotope changes of metabolites associated with amino acid synthesis

**Fig 5:**
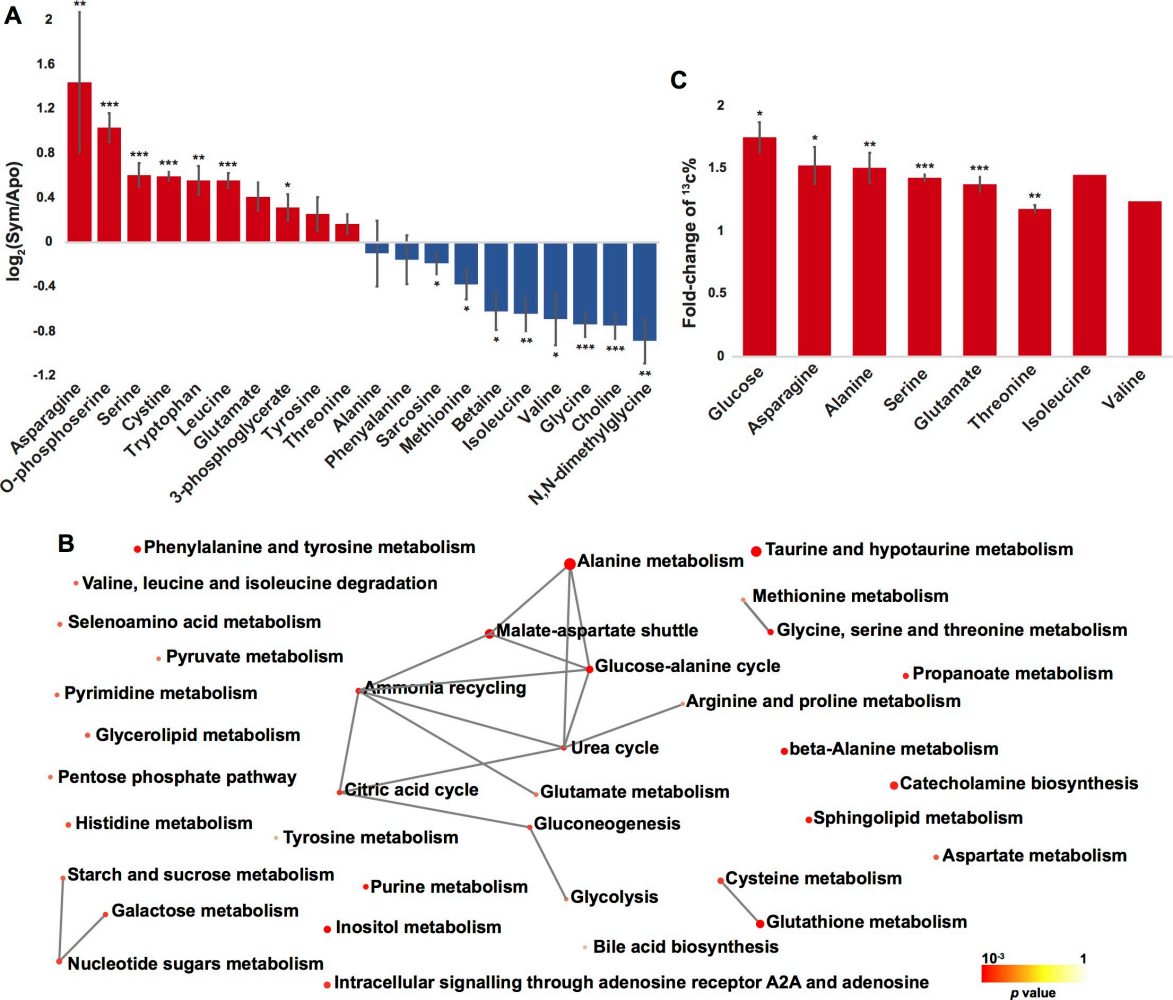
Change of *Aiptasia* metabolite profiles in response to symbiosis. (A) Metabolite abundance changes in response to symbiosis. Color represent abundance changes, with red for increases in symbiotic anemones, blue for increase in aposymbiotic animals. (B) Pathways associated with ^13^C-enriched metabolites. (C) Increasing ^13^C proportion of glucose and amino acids in symbiotic *Aiptasia*. Asterisks denote statistical significance of the changes (two-tail *t* test: * *p* < 0.05, ** *p* < 0.01, *** *p* < 0.001). Statistical testing of isoleucine and valine was not possible as they were detected in only one aposymbiotic replicate with reasonable concentration. Error bar represents standard error of the mean.

We first investigated metabolomes of symbiotic and aposymbiotic anemones using nuclear magnetic resonance (NMR) spectroscopy. Three metabolites in the *de novo* serine biosynthesis pathway were highly abundant in symbiotic *Aiptasia* (two of them significantly so, *p* < 0.05), while five out of the six intermediates in the alternative glycine/serine biosynthesis pathway using food-derived choline were significantly enriched in aposymbiotic anemones as predicted (Fig 4B, Fig 5A). However, as glucose produces multiple peaks in the ^1^H NMR spectrum, and most of these peaks overlap with many other potential metabolites in both symbiotic and aposymbiotic anemones, it was not possible to precisely determine glucose concentrations via NMR. Consequently, we performed ^13^C bicarbonate labeling experiments and compared metabolite profiles of symbiotic and aposymbiotic anemones using gas chromatography-mass spectrometry (GC-MS), in order to test if the glucose is indeed provided by the symbiont and if the downstream usage of symbiont derived organic carbon is in the host. Our experiments confirmed that symbionts provide large amounts of ^13^C-labeled glucose to the host (S4 Fig) and that the ^13^C-labeling was significantly enriched in many amino acids and their precursors in symbiotic anemones compared to aposymbiotic ones (S5 Table). Moreover, metabolite set enrichment analysis indicated that ^13^C was mainly enriched in several amino acid metabolism pathways (Fig 5B), which is consistent with the enrichment analysis of the 731 differentially expressed core-symbiosis genes. For the amino acids with good abundance in both symbiotic and aposymbiotic animals, we examined the proportion of ^13^C in each of them, respectively. Interestingly, we observed relatively stable increases (~1.5-fold) of ^13^C levels in symbiotic animals compared to aposymbiotic ones (Fig 5C). This constant increase suggests a single carbon source (photosynthesis-produced glucose) rather than multiple sources (glucose and symbiont-derived amino acids) involved in host amino acid biosynthesis. In the latter case, we would expect to identify more amino acid transporter genes being differentially expressed in response to symbiosis, which is not the case according to the meta-analysis. This provides further proof for symbiont derived glucose as the carbon source used for host amino acid synthesis.

## Discussion

### Meta-analysis in the identification of symbiosis-associated genes

Technical variation during experimentation may introduce strong bias in high throughput sequencing studies [21]. This is especially true in the study of symbiotic systems. Since such systems usually feature highly interdependent metabolic interactions, technical variations in culturing, sampling, and/or manipulation can be expected to introduce significant noise in the metabolic processes intertwined with real symbiosis-associated signals. However, this is often overlooked in transcriptomic studies, and especially so in non-model organisms. As we have noticed, the non-experimental parameters sometimes exerted stronger effects on the expression profiles than the symbiotic state in the *Aiptasia* transcriptomic studies. To reduce the high signal-to-noise ratio, we suggest two potential venues for differential expression studies. Firstly, future transcriptomic efforts should take extreme care to standardize all experimental conditions save for the one under study. For example, culture conditions should be identical across treatments except for the factor under study, treatments should further be performed on multiple independent batches, RNA extractions and library preparation should be carried out on all samples simultaneously. The prepared libraries should also be sequenced on the same run to further minimize technical variation. Secondly, one should not dogmatically adhere to the convention of using *p* = 0.05 as the cutoff for statistical significance. If a study considers one in every three genes as significantly differentially expressed, to a careful reader, the proclaimed significance of those genes is diminished. As the number of DEGs increase, the rate of type I errors would also increase, which makes the discovery of meaningful biological processes more difficult.

Consequently, meta-analyses have become a powerful approach for summarizing sequencing data from different trials in order to reduce the biases inherent to single experiments and to increase the statistical power for the identification of underlying common processes [15, 16]. By applying this approach to *Aiptasia* RNA-seq data, we were able to deal with the specific variances present in the individual studies, improve the precision in effect size estimation for each of the genes, and eventually identify a group of high-confidence symbiosis-associated candidates. As shown in our Monte Carlo simulations of PVCA, the genes we identified using meta-analysis exhibited significantly higher association with symbiotic state than any of the single experiment analyses. Moreover, the functional enrichment analyses of our core gene set presented more symbiosis-related GO terms and KEGG pathways, rather than the very broad terms identified from individual studies, being enriched. These terms were also enriched significantly with relatively smaller *p* values in meta-analysis-identified genes and assisted in the understanding of metabolic interactions between host and symbiont.

### Metabolic equilibrium in cnidarian-Symbiodiniaceae symbiosis

**Fig 6:**
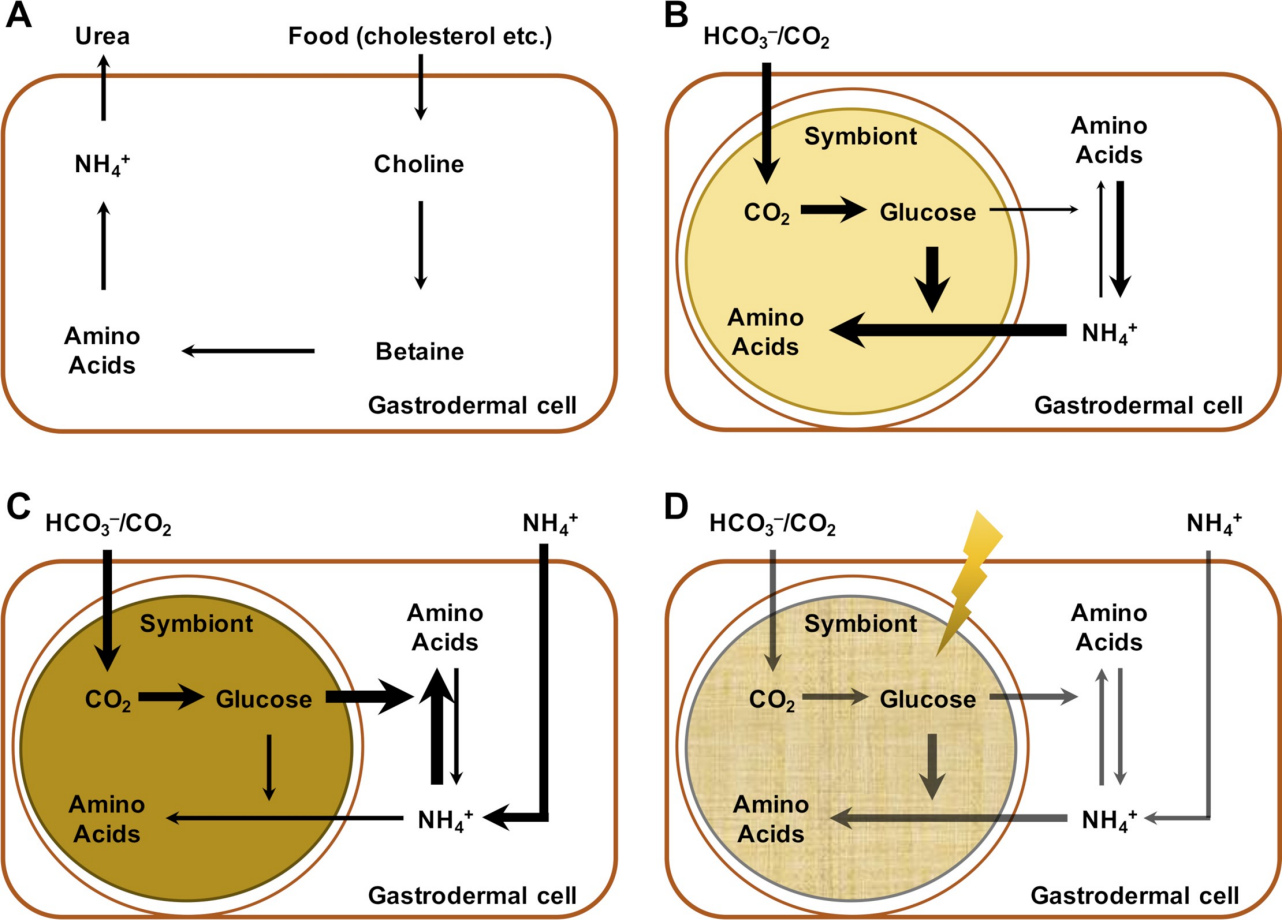
A predicted model of metabolic interactions between host and symbiont at different stages. (A) Aposymbiotic stage. Host cells synthesize amino acids from food-derived choline. The ammonium produced from the catabolism of amino acids is converted to urea and evacuated from host cells. (B) Early symbiotic stage. The animal host is partially colonized by symbiont cells which produce glucose via photosynthesis. A large proportion of the glucose remains in the symbiont for its own proliferation, while a minimum amount of glucose is transferred to host and enables host-dependent ammonium assimilation. The production of amino acids frees host from its dependence on food-derived choline. (C) Fully symbiotic stage. The proliferation of symbiont cells requires more nitrogen to produce more glucose. This further enhances the capability of the host to assimilate ammonium and control the nitrogen availability to symbiont. The process reaches an equilibrium eventually where symbiont cell density is stable. (D) Stressed stage. The balance of this system depends on the photosynthesis of symbiont and the translocation of photosynthates to the host. Stresses, such as temperature stress imposed by climate change, reduce photosynthetic efficiency of the symbionts and photosynthate translocation, resulting in reduced ammonium assimilation by the host and increased ammonium availability to the symbionts. The increased availability of nitrogen to the symbionts can induce cell proliferation and further reduction of photosynthate translocation which exacerbates the metabolic imbalance of the system and initiates a vicious cycle.

The metabolic interactions between host and symbionts have been of great interest in the study of the cnidarian-Symbiodiniaceae symbiosis [22, 23]. Among these interactions, the exchange of carbonic and/or nitrogenous compounds between the two partners is arguably a central process that has been the focus of many investigations [5, 7, 9, 24–26]. However, the connections between these two major compounds remains unclear and highly controversial. By combining a meta-analysis of transcriptomic data, metabolomics, and ^13^C profiling, we demonstrated a host-dependent negative feedback mechanism in the regulation of nitrogen availability to the symbionts, which is driven by symbiont-derived fixed carbon (Fig 6).

The systematic upregulation of genes involved in choline-betaine pathway highlights the heterotrophic state of aposymbiotic *Aiptasia* (Fig 6A). This also emphasizes the importance of regular feeding in the maintenance of aposymbiotic animals as previously stated [27]. The downregulation of choline transport in symbiotic *Aiptasia* indicates a decrease of the host’s demand on dietary choline (Fig 6B and 6C). Correspondingly, genes involved in the downstream conversion of choline to betaine and the production of glycine from betaine are also downregulated. The decrease of glycine caused by this downregulation is likely compensated by the metabolism of serine, which can be achieved by the observed upregulation of serine hydroxymethyltransferase (SHMT, AIPGENE4781), which catalyzes the interconversion of glycine and serine. Interestingly, our results suggest that serine is one of the key components in amino acid interconversion, as the genes involved in its *de novo* biosynthesis from 3-phosphoglycerate (one of the intermediates of glycolysis) were consistently upregulated. The conversion from glutamate to 2-oxoglutarate, catalyzed by the upregulated phosphoserine aminotransferase (PSAT, AIPGENE17104), may serve as the main reaction to provide amino groups for the biosynthesis of amino acids. Since 2-oxoglutarate is also one of the intermediates in the citrate acid cycle, an increase of glucose provided by the symbionts may also increase the overall activity of the cycle, hence raising the relative abundance of 2-oxoglutarate in symbiotic animals. High levels of 2-oxoglutarate have been reported to induce ammonium assimilation through glutamine synthetase / glutamate synthase cycle in bacteria [28]. Consistent with this finding, we observed upregulation of all the genes involved in this pathway for symbiotic anemones.

Metabolomic analyses of symbiotic and aposymbiotic anemones confirmed the predictions derived from our transcriptomic meta-analysis. Most of the metabolic intermediates in the *de novo* serine biosynthesis using symbiont-derived glucose were highly enriched in symbiotic anemones and showed increased ^13^C-labeling. Conversely, many of the metabolites from choline-betaine-glycine-serine conversion showed decreased abundance in symbiotic animals. Furthermore, we also identified many other amino acids with significantly increased abundance and ^13^C-labeling signals, suggesting that serine may serve as a metabolic intermediate for the production of other amino acids. Overall, these results highlight that symbiont-derived glucose fuels ammonium assimilation and amino acid production in the host and that serine biosynthesis acts as a main metabolic hub in symbiotic hosts (Fig 6B and 6C).

The strong shifts in host amino acid metabolic pathways induced by symbiont-provided glucose explains the interactions between nitrogen and carbon metabolism in the *Aiptasia*-Symbiodiniaceae symbiosis. The catabolism of glucose through pathways such as glycolysis, pentose phosphate pathway, and citric acid cycle, not only generates more energy (in forms of ATP, NADH, and NADPH), which is critical to ammonium assimilation, but also produces more intermediate metabolites that can serve as carbon backbones in many biosynthetic pathways such as amino acid synthesis. Our findings thus highlight nitrogen conservation, i.e. the host driven assimilation of waste ammonium using symbiont-derived carbon, as a central mechanism of the cnidarian-algal endosymbiosis [7]. This metabolic interaction might serve as a self-regulating mechanism for the host to control symbiont density through the regulation of nitrogen availability [5] in a carbon dependent manner. This allows for higher nitrogen availability in the early stages of infection (few symbionts translocating little carbon and requiring little nitrogen) and gradual reduction of nitrogen availability with increasing symbiont densities (many symbionts translocating more carbon and requiring more nitrogen). The strict dependence of this mechanism on symbiont-derived carbon highlights the sensitivity of this relationship to changes in carbon translocation from the symbiont to the host as imposed by environmental stresses (Fig 6D). Heat-challenged symbionts have been shown to retain significantly more carbon for their own proliferation using the increased nitrogen availability [29], while exhibiting a significant decline in light utilization efficiency [30]. This indicates that the balance of the negative-feedback system is tipped by climate change-induced heat stress, because such stress disrupts carbon translocation from the symbionts to the host while increasing the amount of nitrogen available to the symbionts. Overall, this sensitive metabolic equilibrium presents a potential molecular mechanism underlying the establishment, maintenance, and breakdown of symbiotic relationships between cnidarian hosts and Symbiodiniaceae.

## Materials and methods

### Data collection and pre-processing

To collect data for a meta-analysis, we screened for transcriptomic study that focused on cnidarian-Symbiodiniaceae symbiosis using the clonal *Aiptasia* strain CC7. We obtained 3 previous RNA-seq studies that met our criteria and provided 4 separate datasets [11–13]. All the datasets were generated on the same platform (Illumina HiSeq 2000). Three of the datasets contained 101 bp paired-end reads, while the last one contained 36 bp single-end reads. Samples were labeled based on the initials of the first author of published papers: YL, SB, EML, EML-36.

As all raw data from Lehnert et al. [11] was provided as a monolithic FASTQ file, a custom Python script was written to split the reads into its constituent replicates, as inferred from the FASTQ annotation lines.

### Identification of DEGs

To avoid biases stemming from the use of disparate bioinformatics tools in calling DEGs, data from the four datasets were processed with identical analytical pipelines.

Gene expressions were quantified (in TPM, transcripts per million) based on the published *Aiptasia* gene models [12] using kallisto v0.42.4 [31]. DEGs were independently identified in the four datasets using sleuth v0.28.0 [32]. Genes with corrected *p* values < 0.05 were considered differentially expressed.

To enable direct comparisons of gene expression values between datasets, another normalization with sleuth was carried out on all samples (*n* = 17 aposymbiotic and *n* = 17 symbiotic). Principal component analysis (PCA) and ranked correlation analysis (RCA) were carried out on these normalized expression values to assess the relationship between samples and reproducibility of these studies.

### Profiling sources of batch effects

Principal variance components analysis (PVCA), a technique that was developed to estimate the extent of batch effects in microarray experiments [17], was used several times in our study. A PVCA was carried out on raw data to estimate the batch effects in the combined dataset and their possible source in the original experimental designs. Consistently, the normalized data was also assessed for the reduction of batch effects post-normalization. We also performed PVCA on normalized expression values of the DEGs identified in each independent analysis or the final meta-analysis to detect the robustness of DEG calling.

### Meta-analysis across studies

For every gene with at least two studies with significant differential expression values, a meta-analysis was performed to determine the overall effect size and associated standard error. Effect sizes from each study *i* (represented as *w*_*i*_) were calculated as the natural logarithm of its expression ratio (ln *R*_*i*_), i.e. geometric means of all expression values in the aposymbiotic state divided by the geometric means of all expression values in the symbiotic state. Conveniently, this value is approximately equal to the *β*_*i*_ value provided by sleuth. As sleuth also calculates the standard error of *β*_*i*_, the variance of ln *R*_*i*_ was not calculated via the typical approximation—instead, the variance *v*_*i*_ was directly calculated as

$$v_{i}={SE}_{\beta_{i}}^{2}∙n_{i}$$

where *n*_*i*_ represents the number of replicates in study *i*.

To combine the studies, a random-effects model was used. While the use of this model is somewhat discouraged for meta-analyses with few studies as it is prone to produce type I errors [33], we still opted for its use over the fixed-effects model due to the substantial inter-study variation evident in the PCAs performed previously. Also, the type I error rate could be controlled by setting a more conservative *p* threshold, if required.

The DerSimonian and Laird [14] method was implemented as described below. Studies with individual effect sizes *m*_*i*_ were weighted (w*) by a combination of the between-study variation (*τ*^2^) and within-study variation (*v*_*i*_), according to the formula

$$w_{i}^{*}=\frac{1}{v_{i}+\tau^{2}}$$

The between-study variation (*τ*^2^) across all *k* studies was calculated as

$$\tau^{2}=\max\left\{\frac{Q-df}{C},0\right\}$$

where

$$Q=\sum {w_{i}(T_{i}-\bar{T})}^{2}$$

$$C=\sum w_{i}-\frac{\sum w_{i}^{2}}{\sum w_{i}}$$

The weighted mean (m*) was calculated as

$$m^{*}=\frac{\sum w_{i}^{*}T_{i}}{\sum w_{i}^{*}}$$

while the standard error of the combined effect was

$$SE(m^{*})=\frac{1}{\sqrt{\sum w_{i}^{*}}}$$

The two-tailed p-value was calculated using

$$p=2\left[1-\Phi (\left|\frac{m^{*}}{SE(m^{*})}\right|)\right]$$

and then subsequently corrected for multiple hypothesis testing with the Benjamini-Hochberg-Yekutieli procedure [34, 35] using a Python script. Genes with corrected *p* < 0.05 were considered differentially expressed. For transparency, calculations for all equations were implemented manually in Microsoft Excel (S1 Table) following established guidelines [36].

### Functional interpretation of DEGs

Gene ontology (GO) and KEGG pathway enrichment analyses were both conducted on five DEG lists: one each from the four independent datasets, and one from the results of the meta-analysis.

Identification of enriched GO terms were conducted using topGO [37] by a self-developed R script (https://github.com/lyijin/topGO_pipeline). A GO term was considered enriched only when its *p* value was less than 0.05.

KEGG pathway enrichment analyses were performed using Fisher’s exact and subsequent multiple testing correction via false discovery rate (FDR) estimation. A KEGG pathway was deemed enriched (or depleted) only when its FDR was less than 0.05. The results of enrichment analyses were visualized using GOplot [38].

### Metabolomic profiles of symbiotic and aposymbiotic anemones

Aposymbiotic *Aiptasia* strain CC7 and the same strain in symbiosis with *Breviolum minutum* strain SSB01 (formerly *Symbiodinium minutum* SSB01) [1] were used for metabolic profiling. All the symbiotic and aposymbiotic anemones were maintained in the laboratory in autoclaved seawater (ASW) at 25°C in 12-hour light/12-hour dark cycle with light intensity of ~30 μmol photons m^-2^s^-1^ for over three years. Anemones were fed three times a week with freshly hatched *Artemia* nauplii, and water change was done on the day after feeding.

Anemones were rinsed extensively to remove any external contaminations, and starved for two days in ASW and transferred into ASW with 10 mM ^13^C-labelled sodium bicarbonate (Sigma-Aldrich, USA) for another two days before sampling. The four-day starvation period ensured all *Artemia* had been digested and consumed, hence there was no contamination from *Artemia* in the samples for NMR and GC-MS. The samples were drained completely on clean tissues to remove any water on surface, then snap frozen in liquid nitrogen to avoid any further metabolite changes in downstream processing.

To compare metabolomic profiles of anemones at different symbiotic states, four replicates of each state (*n* = 30 individuals per replicate), were processed for metabolite extraction using a previously reported methanol/chloroform method [39]. The free amino acid-containing methanol phase was dried using CentriVap Complete Vacuum Concentrators (Labconco, USA).

For NMR metabolite profiling, samples were dissolved in 600 μl of deuterated water (D_2_O), and vortexed vigorously for at least 30 seconds. Subsequently, 550 μL of the solution was transferred to 5 mm NMR tubes. NMR spectrum was recorded using 700 MHz AVANCE III NMR spectrometer equipped with Bruker CP TCI multinuclear *CryoProbe* (BrukerBioSpin, Germany). To suppress any residual HDO peak, the ^1^H NMR spectrum were recorded using excitation sculpting pulse sequence (zgesgp) program from Bruker pulse library. To achieve a good signal-to-noise ratio, each spectrum was recorded by collecting 512 scans with a recycle delay time of 5 seconds digitized into 64 K complex data points over a spectral width of 16 ppm. Chemical shifts were adjusted using 3-trimethylsilylpropane-1-sulfonic acid as internal chemical shift reference. Before Fourier transformations, the FID values were multiplied by an exponential function equivalent to a 0.3 Hz line broadening factor. The data was collected and quantified using Bruker Topspin 3.0 software (Bruker BioSpin, Germany), with metabolite-peak assignment using Chenomx NMR Suite v8.3, with an up-to-date reference library (Chenomx Inc., Canada).

For ^13^C-labelling investigation using GC-MS, dried samples were re-dissolved in 50 μl of Methoxamine (MOX) reagent (Pierce, USA) at room temperature and derivatized at 60°C for one hour. 100 μl of *N*,*O*-*bis*-(trimethylsilyl) trifluoroacetamide (BSTFA) was added and incubated at 60°C for further 30 min. 2 μl of the internal standard solution of fatty acid methyl ester (FAME) were then spiked in each sample and centrifuged for 5 min at 10,000 rpm. 1 μl of the derivatized solution was analyzed using single quadrupole GC-MS system (Agilent 7890 GC/5975C MSD) equipped with EI source at ionization energy of 70 eV. The temperature of the ion source and mass analyzer was set to 230°C and 150°C, respectively, and a solvent delay of 9.0 min. The mass analyzer was automatically tuned according to manufacturer’s instructions, and the scan was set from 35 to 700 with scan speed 2 scans/s. Chromatography separation was performed using DB-5MS fused silica capillary column (30m x 0.25 mm I.D., 0.25 μm film thickness; Agilent J&W Scientific, USA), chemically bonded with 5% phenyl 95% methylpolysiloxane cross-linked stationary phase. Helium was used as the carrier gas with constant flow rate of 1.0 ml min^-1^. The initial oven temperature was held at 8°C for 4 min, then ramped to 300°C at a rate of 6.0°C min^-1^, and held at 300°C for 10 min. The temperature of the GC inlet port and the transfer line to the MS source was kept at 200°C and 320°C, respectively. 1 μl of the derivatized solution of the sample was injected into split/splitless inlet using an auto sampler equipped with 10 μl syringe. The GC inlet was operated under splitless mode. Metabolites in all samples were identified using Automated Mass Spectral Deconvolution and Identification System software (AMDIS) with the NIST special database 14 (National Institute of Standards and Technology, USA). The mass isotopomer distributions (MIDs) of all compounds were detected and their ^13^C-labelling enrichment in symbiotic *Aiptasia* were investigated using MIA [40]. Pathways associated with these ^13^C-enriched metabolites were explored using MetaboAnalyst v3.0 [41].

## Supporting information

S1 Fig Relationship between symbiotic and aposymbiotic *Aiptasia* in different studies. (A) YL, (B) SB, (C) EML, and (D) EML-36. Colors represent symbiotic states of the samples with blue for animals at aposymbiotic state and orange for symbiotic anemones.
(TIF)

S2 Fig (A) Gene ontology and (B) KEGG pathway enrichment of differentially expressed genes identified from four individual experiments.
(TIF)

S3 Fig KEGG nitrogen metabolism reference pathway. Color represents the expression changes of the gene in response to symbiosis, where red and blue indicate upregulation and downregulation in symbiotic anemones, respectively. 1.4.1.13/1.4.1.14: glutamate synthase (GS, AIPGNENE26077); 6.3.1.2: glutamine synthetase (GOGAT, AIPGENE26763); 1.4.1.4: glutamate dehydrogenase (GDH, AIPGENE24026, AIPGENE27618).
(TIF)

S4 Fig Glucose abundance in *Aiptasia* determined by GC-MS. Lines represent the GC-MS spectrums of anemones at different symbiotic states, with blue for aposymbiotic, and red for symbiotic animals. The peak located between the dotted lines is for glucose.
(TIF)

S1 Table Random effects meta-analysis across four independent datasets. Note: qval, b, seb_b, and var_b are the values extracted from Sleuth, which indicate the statistical significance and level of differential expression of the corresponding gene. up/down indicates upregulation/downregulation of the gene in symbiotic anemones in the comparison with aposymbiotic ones. diff indicates statistically significant expression change, which was assigned when the corresponding qval < 0.05.
(XLSX)

S2 Table Experimental parameters used in each individual study. (XLSX)

S3 Table GO terms enriched by symbiosis-associated genes identified from meta-analysis. (XLSX)

S4 Table KEGG pathways enriched by symbiosis-associated genes identified from meta-analysis. (XLSX)

S5 Table 13C-enriched metabolite identified by MIA from GC-MS data. (XLSX)

S1 Text Supplementary results and discussion. (DOCX)
