# Supplementary figures and images for: Host-dependent nitrogen recycling as a mechanism of symbiont control in Aiptasia
Source: PLoS Genet. 2019 Jun 24;15(6):e1008189. doi: 10.1371/journal.pgen.1008189 (PMC6611638; doi:10.1371/journal.pgen.1008189)

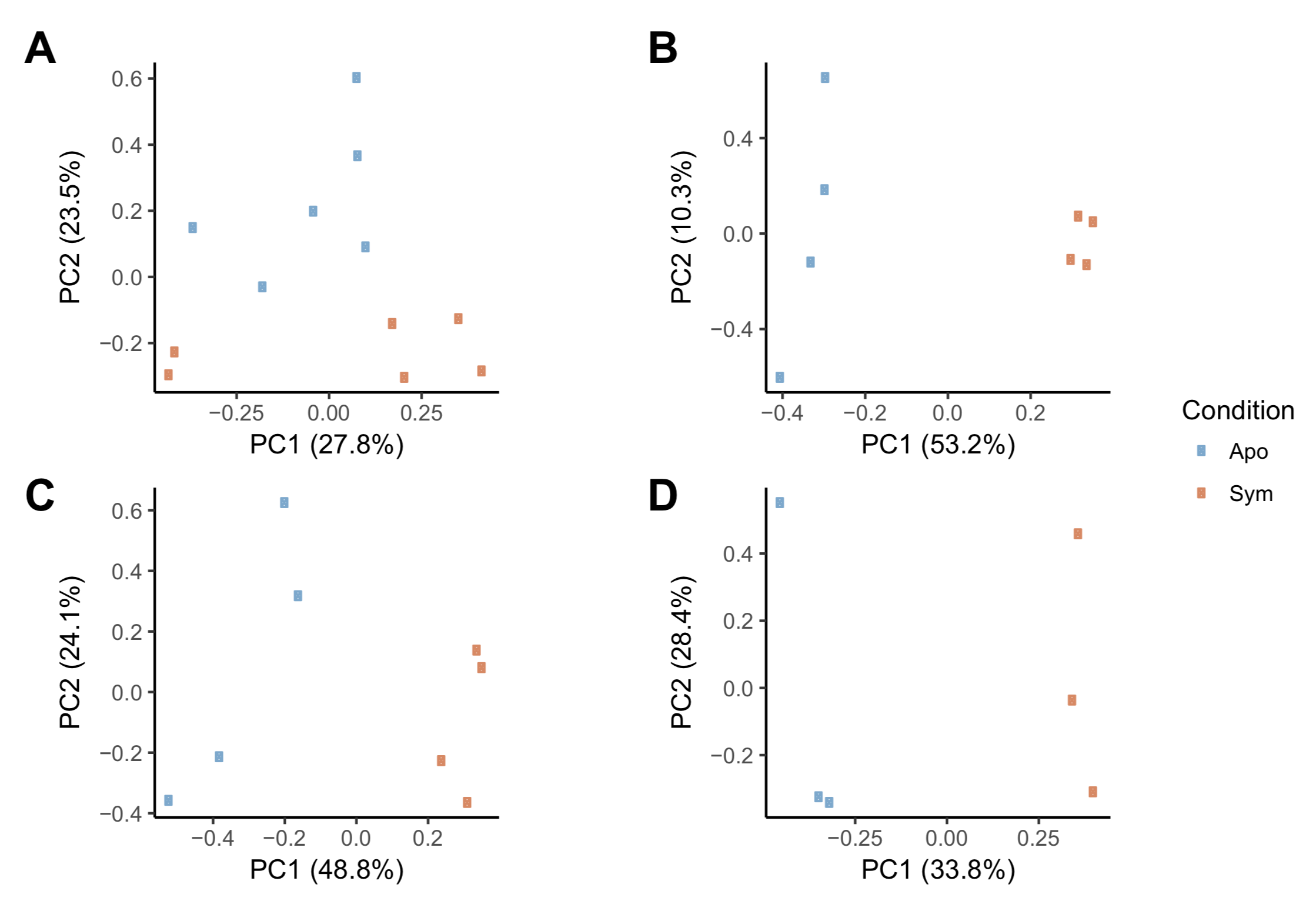

Supplement: S1 Fig — (A) YL, (B) SB, (C) EML, and (D) EML-36. Colors represent symbiotic states of the samples with blue for animals at aposymbiotic state and orange for symbiotic anemones. (TIF) [file pgen.1008189.s001.tif]

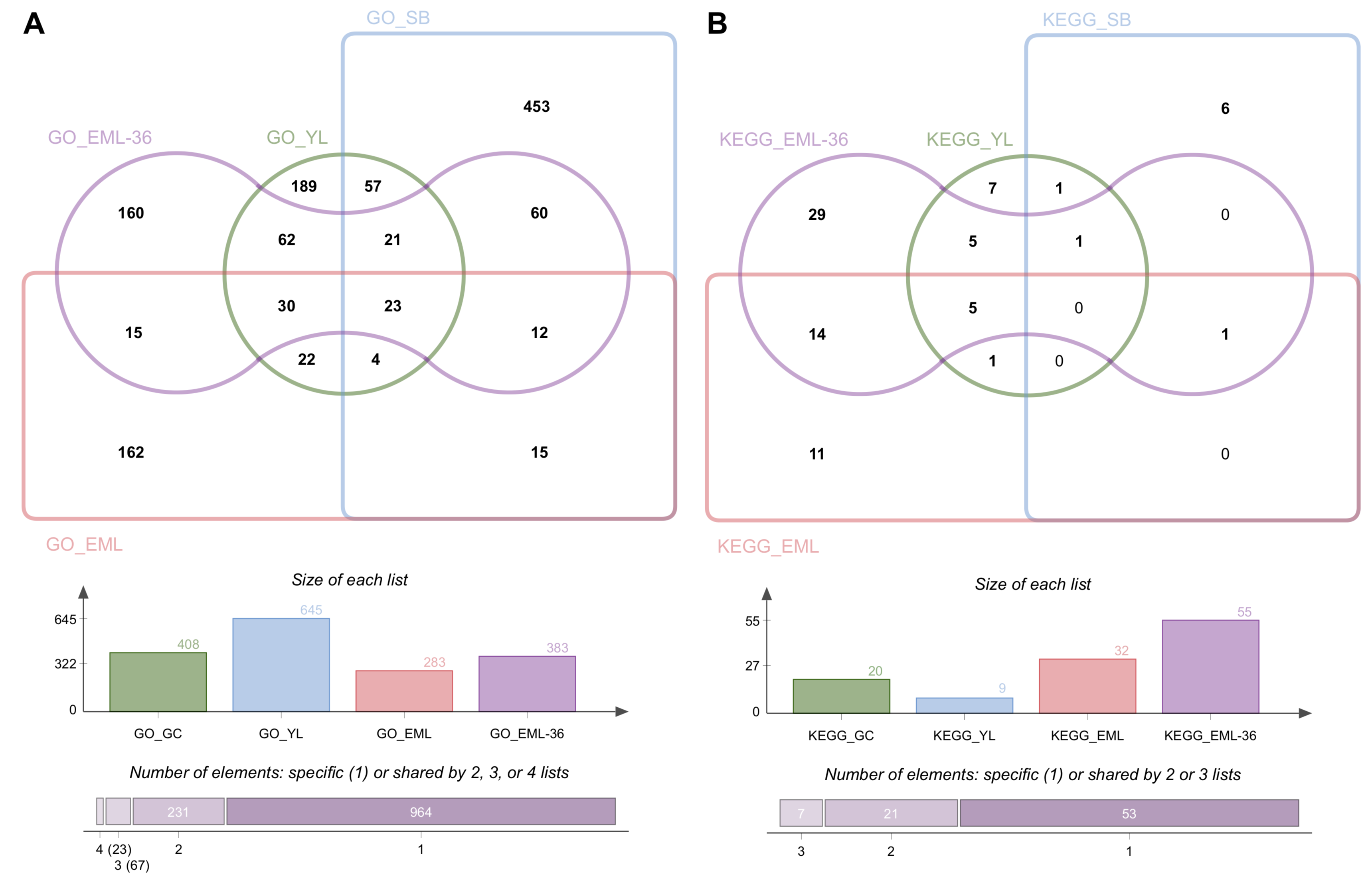

Supplement: S2 Fig — (A) Gene ontology and (B) KEGG pathway enrichment of differentially expressed genes identified from four individual experiments. (TIF) [file pgen.1008189.s002.tif]

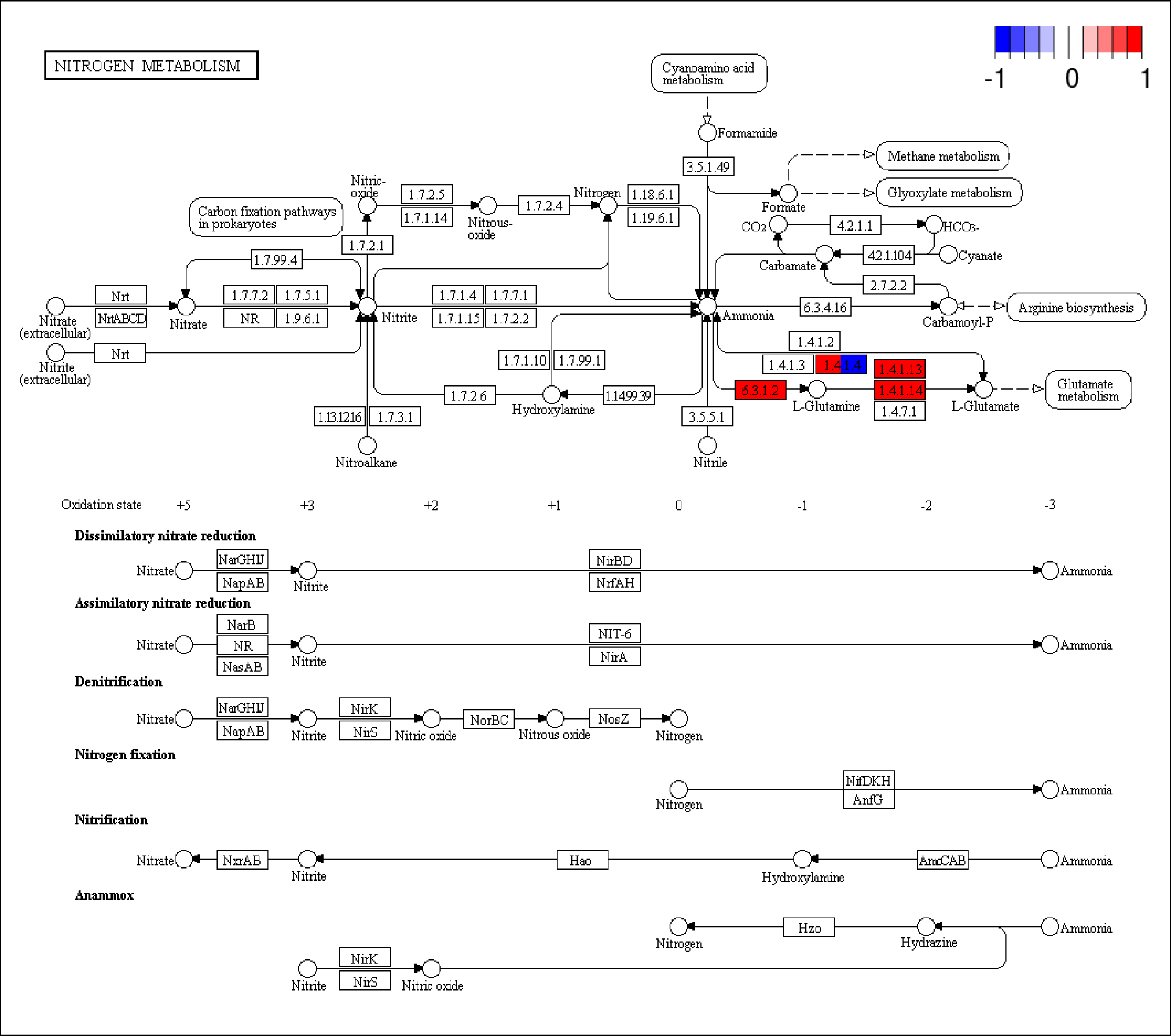

Supplement: S3 Fig — Color represents the expression changes of the gene in response to symbiosis, where red and blue indicate upregulation and downregulation in symbiotic anemones, respectively. 1.4.1.13/1.4.1.14: glutamate synthase (GS, AIPGNENE26077); 6.3.1.2: glutamine synthetase (GOGAT, AIPGENE26763); 1.4.1.4: glutamate dehydrogenase (GDH, AIPGENE24026, AIPGENE27618). (TIF) [file pgen.1008189.s003.tif]

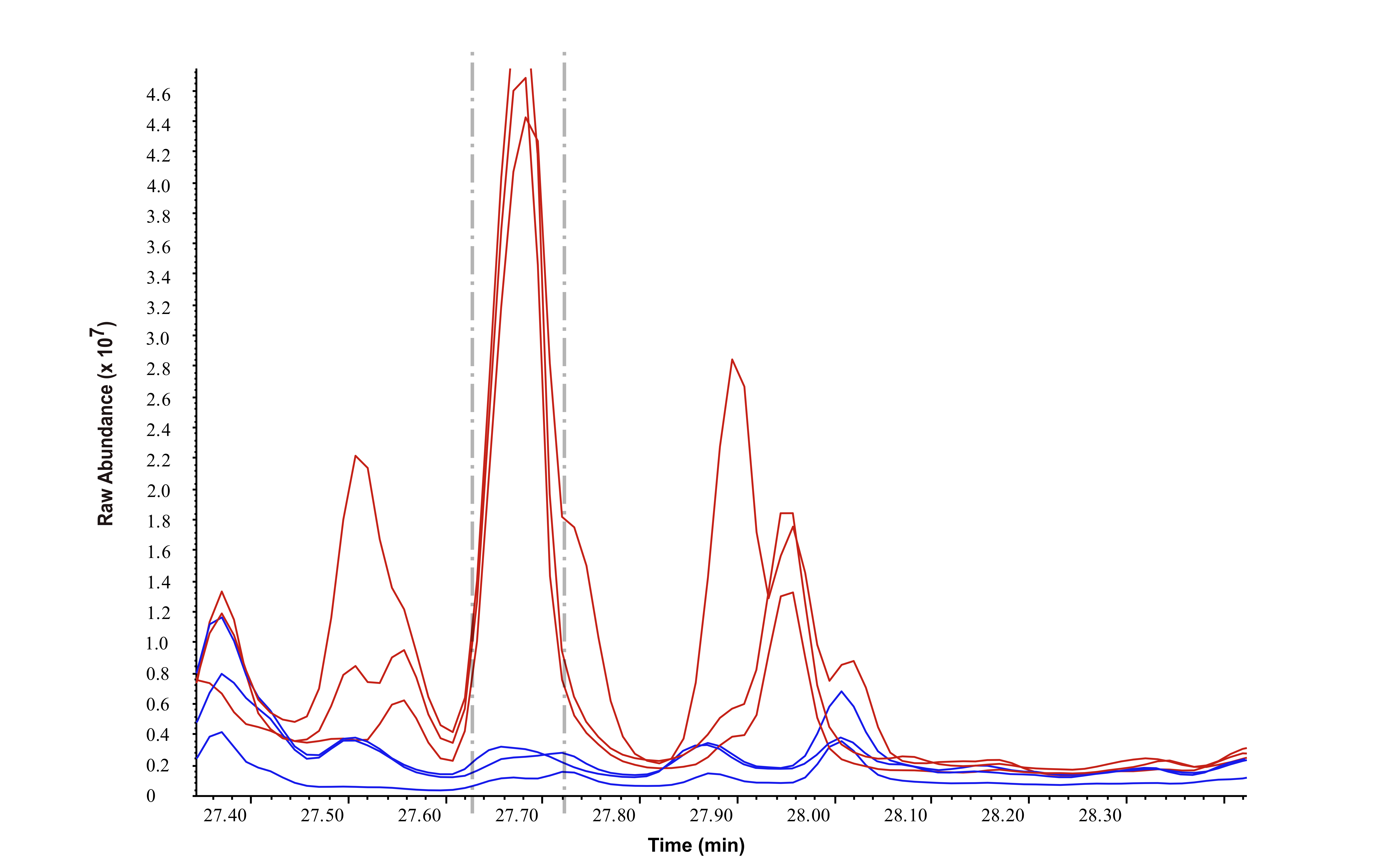

Supplement: S4 Fig — Lines represent the GC-MS spectrums of anemones at different symbiotic states, with blue for aposymbiotic, and red for symbiotic animals. The peak located between the dotted lines is for glucose. (TIF) [file pgen.1008189.s004.tif]
