# Supplementary material for: Host-dependent nitrogen recycling as a mechanism of symbiont control in Aiptasia
Source: PLoS Genet. 2019 Jun 24;15(6):e1008189. doi: 10.1371/journal.pgen.1008189 (PMC6611638; doi:10.1371/journal.pgen.1008189)
Supplement: S1 Text — (DOCX) [file pgen.1008189.s010.docx]

### S1 Text: Supplementary Results and Discussion

To disentangle the gene expression information associated with *Aiptasia*-*Symbiodinium* symbiosis, we collected the published symbiosis-centered *Aiptasia* RNA-Seq data, performed differential expression analysis on each of the collected datasets using the exact same pipeline, then applied random-effects meta-analysis on the outputs of the independent differential analyses. Eventually, we identified total 731 genes differentially expressed across the published data. Functional interpretation of these genes reveals tight association with many symbiosis-related processes, as previously reported over the last two decades [*1*–*9*]. However, we have narrowed the genes associated with these processes down to a reasonable range compared to previous publications. Here, we provide a summary of the functions of these genes based on the pathways they engage in.

#### Host-symbiont recognition and symbiosis establishment

The establishment of the endosymbiosis requires several key steps that involve the recognition of the symbiont by host immune system, the phagocytotic uptake of the symbiont, and the likely suppression of host apoptotic responses. We identified several GO terms and KEGG pathways involved in these processes that were significantly enriched.

The recognition of symbionts by the host cell is thought to be the initial step of symbiosis establishment [*10*, *11*]. In line with that, we found that many genes related to cell recognition and cell adhesion during phagocytosis were differentially expressed in response to symbiosis (Table SS1). The interaction between host pattern-recognition receptors and symbiont microbe-associated molecular patterns is believed to be the first step of the recognition process which triggers the activation of the host innate immune system and subsequent phagocytosis [*10*–*12*]. Consistently, we found that a secreted lectin, ficolin-1, was significantly upregulated in symbiotic animals, while a gene from another type of lectin, techylectin-5B, and a gene encoding fibrinogen C domain-containing protein 1 (FIBCD1) were downregulated. These proteins were previously reported to be involved in interactions with the acetylated compounds on microbial cell surface, and endocytosis [*13*–*17*]. Interestingly, glycans containing N-acetyl groups have been detected on the cell surface of a broad range of *Symbiodinium* strains [*18*, *19*]. This may indicate that the interactions between these differentially expressed genes and acetylated microbe-associated molecular patterns on *Symbiodinium* cell surface are the potential initial recognitions. The involvement of ficolin-1, or techylectin-5B and FIBCD1 may lead, respectively, to accept competent symbionts, or to reject non-competent strains. Moreover, the upregulation of genes associated with cell adhesion, collagen alpha-2(VI) chain (COL6A2) [*20*], and integrin alpha-8 (ITGA8) [*21*], may also indicate the genes involved in the downstream engulfment of symbiont after host-symbiont recognition.

Following the activation of the innate immune system, programmed cell death has been observed as a common response to pathogen infection in a wide range of animals and plants [*22*, *23*]. The large number of differentially expressed apoptosis-related genes in our study indicates that apoptosis is actively involved in the establishment and maintenance of symbiosis. Apoptosis-inducing factor 2 (AIF2) [*24*], cleft lip and palate transmembrane protein 1 (CLPTM1) [*25*], and tumor necrosis factor ligand superfamily member 10 (TNFSF10) [*26*, *27*], which encode proteins that induce apoptosis when they are highly expressed, were all downregulated in the symbiotic animals. Correspondingly, the apoptosis inhibition genes, hypoxia upregulated protein 1 (HYOU1) [*28*], fibroblast growth factor receptor 3 (FGFR3) [*29*, *30*], and serine/threonine-protein phosphatase 5 (PPP5C) [*31*–*33*], were all upregulated in response to symbiosis. This suggests that the apoptotic response is actively suppressed in symbiotic animals, and may only be activated in response to the breakdown of symbiosis. Consistently, we also find several key genes in sphingolipid rheostat, sphingomyelin phosphodiesterase and sphingosine-1-phosphate phosphatase, to be downregulated in the symbiotic animals. The two components in this rheostat, ceramide and sphingosine, favor anti-proliferation and cell death pathways such as senescence and apoptosis, whereas another product, sphingosine-1-phosphate (S1P), has been shown to suppress apoptosis [*34*, *35*], enhance cell survival [*36*], and promote symbiosis stability [*37*]. These gene expression changes imbalance the rheostat and direct the metabolite flux to the production of S1P, which may also indicate that apoptosis is repressed in symbiosis. However, there are some conflicting evidence: two anti-apoptotic genes from the BCL-2 family, BCL2L1 and BCL2L2 [*38*], were differentially expressed in different directions. Further investigation is needed to fully elucidate the functions of these genes in symbiosis.

**Table SS1** **Differential expression of genes associated with establishment and maintenance of symbiosis.** Fold change represents the relative expression change in symbiotic anemones over aposymbiotic animals.

| **Gene ID** | **Annotation** | **ln (fold change)** |
| --- | --- | --- |
| **Genes related to cell recognition:** | | |
| AIPGENE607 | Galectin-3-binding protein | 0.74 |
| AIPGENE13930 | Collagen alpha-2(VI) chain | 0.73 |
| AIPGENE7322 | Ficolin-1 | 0.64 |
| AIPGENE6165 | Integrin alpha-8 | 0.44 |
| AIPGENE2084 | von Willebrand factor A domain-containing protein 5A | -0.97 |
| AIPGENE13762 | Fibrinogen C domain-containing protein 1 | -1.06 |
| AIPGENE29161 | Techylectin-5B | -1.11 |
| **Genes associated with apoptosis:** | | |
| AIPGENE651 | Hypoxia up-regulated protein 1 | 1.02 |
| AIPGENE12986 | Bcl-2-like protein 1 | 0.77 |
| AIPGENE1561 | Fibroblast growth factor receptor 3 | 0.76 |
| AIPGENE1216 | Transmembrane protein 214-A | 0.66 |
| AIPGENE14070 | Importin subunit beta-1 | 0.64 |
| AIPGENE20804 | Serine/threonine-protein phosphatase 5 | 0.48 |
| AIPGENE18864 | Cleft lip and palate transmembrane protein 1 homolog | -0.51 |
| AIPGENE4512 | Bcl-2-like protein 2 | -0.57 |
| AIPGENE2150 | Sphingosine-1-phosphate phosphatase 2 | -0.62 |
| AIPGENE10332 | Sphingomyelin phosphodiesterase | -0.71 |
| AIPGENE9932 | Apoptosis-inducing factor 2 | -0.73 |
| AIPGENE11859 | Organic cation transporter protein | -0.85 |
| AIPGENE13889 | Tumor necrosis factor ligand superfamily member 10 | -1.02 |
| **Genes involved in oxidation response:** | | |
| AIPGENE163 | Protein BTG1 | -0.98 |
| AIPGENE19125 | Superoxide dismutase [Cu-Zn] | -1.01 |
| AIPGENE21344 | Catalase | -1.20 |
| AIPGENE3800 | Probable phenylalanine-4-hydroxylase 1 | -1.31 |
| AIPGENE21459 | Catalase | -1.33 |

#### Host tolerance of symbiont

Cnidarians in symbiosis with photosynthetic symbionts have to mitigate the toxic effects of molecular oxygen [*39*, *40*]. However, genes involved in the conventional reactive oxygen species (ROS) responses, e.g., superoxide dismutase (SOD) and catalase, were previously reported as generally downregulated in various symbiotic sea anemone species, including *Anemonia viridis* [*41*], *Anthopleura elegantissima* [*4*], and *Aiptasia* sp [*8*, *9*]. Similar observations were made in our study (Table SS1), suggesting the presence of an alternative ROS defense mechanism in symbiotic cnidarians. Our results indicate that tyrosine, iodide, and a molecular oxygen sensor (Gyc88E) may be part of the mechanism.

The systematic downregulation of genes involved in tyrosine degradation (Figure. SS1A) suggests the possible accumulation of tyrosine in symbiotic animals. Tyrosine, together with iodide, has been proposed to play a role in protection of the moon jelly, *Aurelia aurita*, from oxidative stress by reacting with ROS-oxidized iodine to form monoiodotyrosine, diiodytyrosine, and thyroxin which can be removed from animal cells [*42*]. Consistently, pendrin, a robust iodide transporter that facilitates both iodide influx and efflux in different tissues and cells [*43*, *44*], was upregulated ~2-fold in symbiotic animals. Moreover, Gyc88E, a gene that codes for an atypical soluble guanylate cyclase, was upregulated ~1.9-fold in symbiotic anemones. This protein was firstly reported to function as a molecular oxygen sensor in *Drosophila melanogaster* [*45*]. The differential expression of these genes may indicate that *Aiptasia* is able to detect the oxygen concentration changes and actively remove ROS to overcome the oxidative stress caused by photosynthetic symbionts.

**Figure SS1** **Amino acid metabolic pathways affected by symbiosis-associated genes**: (A) metabolism of sulfur-containing amino acids; (B) catabolic process of phenylalanine and tyrosine. Colors of the genes indicate their differential expression, with red indicating upregulation and blue downregulation in symbiotic anemones, relative to aposymbiotic ones, respectively.

#### Nutrient exchange and metabolism

The nature of metabolic interactions in the cnidarian-Symbiodinium symbiosis makes the exchange of various nutrients especially important. Accordingly, we find the genes involved in the transport and processing of certain metabolites to be highly affected by symbiosis.

##### Lipid transport and metabolism

Lipids are among the most important metabolites and play major roles in the functional symbiosis of cnidarians and *Symbiodinium* [*46*]. They have effects not only on the primary metabolism pathways in both host and symbiont, but also on the nutrient exchange between partners [*46*–*50*]. Consistently, we found a large number of genes differentially expressed in response to symbiosis.

NPC2-coding genes showed the highest expression changes between symbiotic and aposymbiotic animals (Table SS2). NPC2, together with NPC1, facilitate the intracellular transport of sterols [*51*, *52*]. However, unlike NPC2, NPC1-coding genes were not differentially expressed in most of the studies; rather, it showed decreased expression in symbiotic animals in a few cases (Table S1). These results indicated, on one hand, that the sterols acquired from symbionts might be especially important to the host, which is consistent with the fact that *Aiptasia* cannot synthesize cholesterol by itself [*53*]. Because of the important roles of cholesterol, its demand has to be met either from food or by special supply from symbionts. On another hand, it is also possible that the symbiont-derived NPC1, rather than host-originated, functions together with the upregulated NPC2 in cholesterol transport under symbiotic conditions. Also, the consistent downregulation of the enzyme that produces lathosterol, 3-beta-hydroxysteroid-Delta(8),Delta(7)-isomerase, and the upregulation of two enzymes converting lathosterol to cholesterol, lathosterol oxidase and 7-dehydrocholesterol reductase, may indicate that lathosterol instead of cholesterol is the actual molecule being supplied by *Symbiodinium* (Table SS2). Further chromatography and mass spectrometry analyses may be needed to test this hypothesis.

Another enriched lipid-related process is the beta-oxidation of long chain fatty acids. Two of the mitochondrial L-carnitine shuttle components, carnitine O-palmitoyltransferase 1 and carnitine O-palmitoyltransferase 2, were upregulated in symbiotic anemones (Table SS2). The shuttle system is an essential step for mitochondria to uptake long-chain fatty acids for their followed beta-oxidation [*54*, *55*]. Most of the genes involved in the downstream beta-oxidation were upregulated, which may indicate that the whole process is generally upregulated in response to symbiosis.

Furthermore, it seems that symbiotic animals have distinct lipid pools, since they express different sets of lipid transporters under different symbiotic states. Two nose resistant to fluoxetine (NRF) proteins and a steroidogenic acute regulatory-related lipid transfer protein (START) were upregulated in symbiotic animals. NRF-6 is involved in lipid binding and transport in *C. elegans* [*56*, *57*]. The START protein has been reported as a lipid exchange and/or lipid sensing protein in mammalian cells [*58*]. In addition, a putative lipid-droplet surface binding protein was also upregulated in symbiotic animals, which suggests that the animals acquire lipids secreted by symbionts and store them as lipid bodies. This is also consistent with a previous report showing that both host and endosymbiont play important roles in the regulation of lipid biogenesis in coral *Euphyllia glabrescens* [*59*]. On the other hand, the lipid transporter genes upregulated in aposymbiotic animals, such as the spinster homolog protein, cubilin, long chain fatty acids transporter, and nonspecific lipid-transfer protein, showed low cargo specificity or was associated with starvation-induced lysosomal activities in mammalian cells [*60*, *61*]. This may suggest that the major lipid supplies of aposymbiotic anemones are from nonspecific endocytosis and/or lysosomal degradation of microbial particles.

**TABLE SS2** **Differential expression of genes associated with lipid metabolism.** Fold change represents the relative expression change in symbiotic anemones over aposymbiotic animals.

| **Gene ID** | **Annotation** | **ln (fold change)** |
| --- | --- | --- |
| AIPGENE22527 | Protein NPC2 homolog | 6.57 |
| AIPGENE22473 | Epididymal secretory protein E1 | 4.31 |
| AIPGENE21619 | Aldehyde dehydrogenase family 3 member B1 | 3.42 |
| AIPGENE23673 | Lathosterol oxidase | 3.41 |
| AIPGENE12723 | Aldehyde dehydrogenase family 3 member B1 | 2.44 |
| AIPGENE762 | Putative phospholipase B-like 2 | 2.10 |
| AIPGENE11799 | Putative diacyglycerol O-acyltransferase Rv1760 | 2.01 |
| AIPGENE16407 | Nose resistant to fluoxetine protein 6 | 1.63 |
| AIPGENE13786 | Lipid storage droplets surface-binding protein 2 | 1.62 |
| AIPGENE13828 | Long chain acyl-CoA synthetase 8 | 1.61 |
| AIPGENE6490 | 7-dehydrocholesterol reductase | 1.38 |
| AIPGENE25676 | Inositol-3-phosphate synthase 1-A | 1.28 |
| AIPGENE14819 | Delta(5) fatty acid desaturase | 1.27 |
| AIPGENE11861 | StAR-related lipid transfer protein 9 | 1.14 |
| AIPGENE16386 | Nose resistant to fluoxetine protein 6 | 1.12 |
| AIPGENE19729 | Delta(8)-fatty-acid desaturase | 1.03 |
| AIPGENE19728 | Delta(8)-fatty-acid desaturase | 0.98 |
| AIPGENE17789 | Probable fatty acid methyltransferase | 0.88 |
| AIPGENE11862 | StAR-related lipid transfer protein 9 | 0.85 |
| AIPGENE28492 | Hepatocyte nuclear factor 4-gamma | 0.84 |
| AIPGENE21331 | Carnitine O-palmitoyltransferase 1, liver isoform | 0.77 |
| AIPGENE18576 | Carnitine O-acetyltransferase | 0.76 |
| AIPGENE28928 | Probable ethanolamine kinase | 0.73 |
| AIPGENE28493 | Hepatocyte nuclear factor 4-gamma | 0.72 |
| AIPGENE10201 | Carnitine O-palmitoyltransferase 1, liver isoform | 0.62 |
| AIPGENE5658 | Carnitine O-palmitoyltransferase 2, mitochondrial | 0.59 |
| AIPGENE10200 | Carnitine O-palmitoyltransferase 1, muscle isoform | 0.59 |
| AIPGENE27247 | Cholesterol 24-hydroxylase | -0.43 |
| AIPGENE9114 | Protein spinster homolog 1 | -0.49 |
| AIPGENE16898 | Cholesterol 24-hydroxylase | -0.49 |
| AIPGENE19794 | Non-specific lipid-transfer protein | -0.62 |
| AIPGENE10177 | Long-chain fatty acid transport protein 1 | -0.64 |
| AIPGENE18368 | 15-hydroxyprostaglandin dehydrogenase [NAD(+)] | -0.65 |
| AIPGENE3461 | Enoyl-[acyl-carrier-protein] reductase [NADPH] FabL | -0.78 |
| AIPGENE24793 | Gastric triacylglycerol lipase | -0.89 |
| AIPGENE8352 | Methyltransferase-like protein 7A | -0.94 |
| AIPGENE9039 | Rhamnosyl O-methyltransferase | -0.95 |
| AIPGENE24036 | Cubilin | -1.06 |
| AIPGENE27122 | Ethanolamine-phosphate cytidylyltransferase | -1.12 |
| AIPGENE11660 | Patatin-like phospholipase domain-containing protein 2 | -1.13 |
| AIPGENE21474 | Putative lysosomal acid lipase/cholesteryl ester hydrolase | -1.18 |
| AIPGENE10967 | Probable 3-beta-hydroxysteroid-Delta(8),Delta(7)-isomerase | -1.41 |

##### Transport of inorganic and photosynthetically fixed carbon

The photosynthetic substrate CO_2_ can diffuse freely across cellular membranes between host and symbionts. However, to maintain efficient photosynthesis, high concentration of inorganic carbon in the symbiosome is required that cannot be satisfied by diffusion. The carbonic anhydrase (CA), catalyzing the conversion between CO_2_ and bicarbonate, is believed to play an important role to meet this requirement [*62*]. A total of ten CA coding genes were identified in *Aiptasia* genome, but only one of them, AIPGENE2901, was upregulated ~2.3-fold in symbiotic animals. The differential expression of this CA indicates that the host may actively regulate the uptake of inorganic carbon in response to symbiosis.

It has been proposed that glucose is the major photosynthate transferred from *Symbiodinium* to the cnidarian host [*63*, *64*]. However, the genes annotated as glucose transport were not upregulated in our study. Instead, a gene annotated as facilitating trehalose transport (TRET1, AIPGENE18406) was upregulated ~10.52-fold in symbiotic anemones. It is possible that trehalose is the major form of fixed carbon being transferred from symbiont into host. This would be consistent with the report that *Symbiodinium* release trehalose into environment as a chemoattractant for coral larvae [*65*]. However, when we tried to search for AIPGENE18406 homologs in another previously published de novo assembled *Aiptasia* transcriptome [*8*], the best match appeared to be a glucose transporter upregulated in symbiotic anemones. This suggests that the gene model of this gene may represent a truncated or incomplete genome sequence or gene model and, hence, produce an incorrect annotation. In any case, further experiments are needed to unequivocally determine the specificity of the transporter.

##### Amino acid metabolism

Among amino acid interconversion pathways, the genes associated with the *S*-Adenosyl methionine (SAM) cycle, which functions in the conversions of sulfur-containing amino acids, were systematically upregulated, as previously reported [*8*]. In contrast to the general upregulation of the genes involved in the SAM cycle, the genes coding cystathionine beta-synthase (CBS, AIPGENE25097) and cystathionine gamma-lyase (CTH, AIPGENE510), which together catalyze the biosynthesis of cysteine from homocysteine, was downregulated in symbiotic animals (Figure. SS1B). This indicates that unlike the complete dependency of *Acropora digitifera* on its symbionts for cysteine [*66*], *Aiptasia* expresses CBS regardless of symbiotic state. However, the significant downregulation of CBS in symbiotic animals suggests that cysteine pools might be replenished either directly by symbionts through upregulated SLC7A9 (AIPGENE1716, AIPGENE15008), which was reported to be responsible for high-affinity reabsorption of cystine in human kidney tubule [*67*], or by the conversion from serine via the upregulated serine-O-acetyltransferase (cys2, AIPGENE556). Interestingly, the possible provision of cysteine or intermediate metabolites by the symbiont would provide the basis for an evolutionary scenario leading to the suggested loss of central proteins in the cysteine biosynthesis process of the zooxanthellate coral *A. digitifera* [*66*].

#### Reference

1. O. Barneah, Y. Benayahu, V. M. Weis, Comparative Proteomics of Symbiotic and Aposymbiotic Juvenile Soft Corals. *Mar. Biotechnol.* **8**, 11–16 (2006).

2. M. L. DeBoer, D. A. Krupp, V. M. Weis, Proteomic and transcriptional analyses of coral larvae newly engaged in symbiosis with dinoflagellates. *Comp. Biochem. Physiol. Part D Genomics Proteomics*. **2**, 63–73 (2007).

3. J. Kuo, M.-C. Chen, C.-H. Lin, L.-S. Fang, Comparative gene expression in the symbiotic and aposymbiotic Aiptasia pulchella by expressed sequence tag analysis. *Biochem. Biophys. Res. Commun.* **318**, 176–186 (2004).

4. M. Rodriguez-Lanetty, W. S. Phillips, V. M. Weis, Transcriptome analysis of a cnidarian-dinoflagellate mutualism reveals complex modulation of host gene expression. *BMC Genomics*. **7**, 23 (2006).

5. C. R. Voolstra *et al.*, The host transcriptome remains unaltered during the establishment of coral-algal symbioses. *Mol. Ecol.* **18**, 1823–33 (2009).

6. V. M. Weis, R. P. Levine, Differential protein profiles reflect the different lifestyles of symbiotic and aposymbiotic Anthopleura elegantissima, a sea anemone from temperate waters. *J. Exp. Biol.* **199**, 883–92 (1996).

7. I. Yuyama, T. Watanabe, Y. Takei, Profiling Differential Gene Expression of Symbiotic and Aposymbiotic Corals Using a High Coverage Gene Expression Profiling (HiCEP) Analysis. *Mar. Biotechnol.* **13**, 32–40 (2011).

8. E. M. Lehnert *et al.*, Extensive Differences in Gene Expression Between Symbiotic and Aposymbiotic Cnidarians. *G3 Genes|Genomes|Genetics*. **4**, 277–295 (2014).

9. C. A. Oakley *et al.*, Symbiosis induces widespread changes in the proteome of the model cnidarian Aiptasia. *Cell. Microbiol.* **18**, 1009–1023 (2016).

10. A. Z. Poole, S. A. Kitchen, V. M. Weis, The Role of Complement in Cnidarian-Dinoflagellate Symbiosis and Immune Challenge in the Sea Anemone Aiptasia pallida. *Front. Microbiol.* **7**, 519 (2016).

11. S. K. Davy, D. Allemand, V. M. Weis, Cell Biology of Cnidarian-Dinoflagellate Symbiosis. *Microbiol. Mol. Biol. Rev.* **76**, 229–261 (2012).

12. G. Eberl, A new vision of immunity: homeostasis of the superorganism. *Mucosal Immunol.* **3**, 450–460 (2010).

13. S. Gokudan *et al.*, Horseshoe crab acetyl group-recognizing lectins involved in innate immunity are structurally related to fibrinogen. *Proc. Natl. Acad. Sci.* **96**, 10086–10091 (1999).

14. E. Gout *et al.*, Carbohydrate recognition properties of human ficolins: glycan array screening reveals the sialic acid binding specificity of M-ficolin. *J. Biol. Chem.* **285**, 6612–22 (2010).

15. A. Schlosser *et al.*, Characterization of FIBCD1 as an Acetyl Group-Binding Receptor That Binds Chitin. *J. Immunol.* **183**, 3800–3809 (2009).

16. T. Thomsen *et al.*, The Recognition Unit of FIBCD1 Organizes into a Noncovalently Linked Tetrameric Structure and Uses a Hydrophobic Funnel (S1) for Acetyl Group Recognition. *J. Biol. Chem.* **285**, 1229–1238 (2010).

17. J. Zhang *et al.*, Secreted M-Ficolin Anchors onto Monocyte Transmembrane G Protein-Coupled Receptor 43 and Cross Talks with Plasma C-Reactive Protein to Mediate Immune Signaling and Regulate Host Defense. *J. Immunol.* **185**, 6899–6910 (2010).

18. L. K. Bay *et al.*, Infection Dynamics Vary between Symbiodinium Types and Cell Surface Treatments during Establishment of Endosymbiosis with Coral Larvae. *Diversity*. **3**, 356–374 (2011).

19. D. D. K. Logan, A. C. LaFlamme, V. M. Weis, S. K. Davy, Flow-cytometric characterization of the cell-surface glycans of symbiotic dinoflagellates (Symbiodinium spp.). *J. Phycol.* **46**, 525–533 (2010).

20. E. Koller, K. H. Winterhalter, B. Trueb, The globular domains of type VI collagen are related to the collagen-binding domains of cartilage matrix protein and von Willebrand factor. *EMBO J.* **8**, 1073–7 (1989).

21. M. Lu *et al.*, Integrin alpha8beta1 mediates adhesion to LAP-TGFbeta1. *J. Cell Sci.* **115**, 4641–8 (2002).

22. N. S. Coll, P. Epple, J. L. Dangl, Programmed cell death in the plant immune system. *Cell Death Differ.* **18**, 1247–1256 (2011).

23. Y. Weinrauch, A. Zychlinsky, The Induction of Apoptosis by Bacterial Pathogens. *Annu. Rev. Microbiol.* **53**, 155–187 (1999).

24. Y. Ohiro *et al.*, A novel p53-inducible apoptogenic gene, PRG3, encodes a homologue of the apoptosis-inducing factor (AIF). *FEBS Lett.* **524**, 163–171 (2002).

25. K. Yamamoto, A. Okamoto, S. Isonishi, K. Ochiai, Y. Ohtake, A Novel Gene, CRR9, Which Was Up-Regulated in CDDP-Resistant Ovarian Tumor Cell Line, Was Associated with Apoptosis. *Biochem. Biophys. Res. Commun.* **280**, 1148–1154 (2001).

26. R. M. Pitti *et al.*, Induction of Apoptosis by Apo-2 Ligand, a New Member of the Tumor Necrosis Factor Cytokine Family. *J. Biol. Chem.* **271**, 12687–12690 (1996).

27. S. R. Wiley *et al.*, Identification and characterization of a new member of the TNF family that induces apoptosis. *Immunity*. **3**, 673–682 (1995).

28. K. Ozawa *et al.*, 150-kDa Oxygen-regulated Protein (ORP150) Suppresses Hypoxia-induced Apoptotic Cell Death. *J. Biol. Chem.* **274**, 6397–6404 (1999).

29. L. Zhu *et al.*, Fibroblast growth factor receptor 3 inhibition by short hairpin RNAs leads to apoptosis in multiple myeloma. *Mol. Cancer Ther.* **4**, 787–98 (2005).

30. S. Trudel *et al.*, Inhibition of fibroblast growth factor receptor 3 induces differentiation and apoptosis in t(4;14) myeloma. *Blood*. **103**, 3521–8 (2004).

31. M. A. Kutuzov, A. V Andreeva, T. A. Voyno-Yasenetskaya, Regulation of Apoptosis Signal-regulating Kinase 1 (ASK1) by Polyamine Levels via Protein Phosphatase 5. *J. Biol. Chem.* **280**, 25388–25395 (2005).

32. K. Morita *et al.*, Negative feedback regulation of ASK1 by protein phosphatase 5 (PP5) in response to oxidative stress. *EMBO J.* **20**, 6028–36 (2001).

33. V. Ollendorff, D. J. Donoghue, The Serine/Threonine Phosphatase PP5 Interacts with CDC16 and CDC27, Two Tetratricopeptide Repeat-containing Subunits of the Anaphase-promoting Complex. *J. Biol. Chem.* **272**, 32011–32018 (1997).

34. N. C. Hait, C. A. Oskeritzian, S. W. Paugh, S. Milstien, S. Spiegel, Sphingosine kinases, sphingosine 1-phosphate, apoptosis and diseases. *Biochim. Biophys. Acta - Biomembr.* **1758**, 2016–2026 (2006).

35. M. Maceyka, K. B. Harikumar, S. Milstien, S. Spiegel, Sphingosine-1-phosphate signaling and its role in disease. *Trends Cell Biol.* **22**, 50–60 (2012).

36. C. Rutherford *et al.*, Regulation of cell survival by sphingosine-1-phosphate receptor S1P1 via reciprocal ERK-dependent suppression of Bim and PI-3-kinase/protein kinase C-mediated upregulation of Mcl-1. *Cell Death Dis.* **4**, e927 (2013).

37. O. Detournay, V. M. Weis, Role of the Sphingosine Rheostat in the Regulation of Cnidarian-Dinoflagellate Symbioses. *Biol. Bull.* **221**, 261–269 (2011).

38. V. Ayllón, X. Cayla, A. García, A. Fleischer, A. Rebollo, The anti-apoptotic molecules Bcl-xL and Bcl-w target protein phosphatase 1α to Bad. *Eur. J. Immunol.* **32**, 1847–1855 (2002).

39. J. A. Dykens, J. M. Shick, Oxygen production by endosymbiotic algae controls superoxide dismutase activity in their animal host. *Nature*. **297**, 579–580 (1982).

40. S. Richier, P. Furla, A. Plantivaux, P.-L. Merle, D. Allemand, Symbiosis-induced adaptation to oxidative stress. *J. Exp. Biol.* **208**, 277–85 (2005).

41. P. Ganot *et al.*, Adaptations to Endosymbiosis in a Cnidarian-Dinoflagellate Association: Differential Gene Expression and Specific Gene Duplications. *PLoS Genet.* **7**, e1002187 (2011).

42. S. Berking *et al.*, A newly discovered oxidant defence system and its involvement in the development of Aurelia aurita (Scyphozoa, Cnidaria): reactive oxygen species and elemental iodine control medusa formation. *Int. J. Dev. Biol.* **49**, 969–976 (2005).

43. Y. H. Kim *et al.*, Role of pendrin in iodide balance: going with the flow. *Am. J. Physiol. Physiol.* **297**, F1069–F1079 (2009).

44. J. A. Rillema, M. A. Hill, Pendrin transporter carries out iodide uptake into MCF-7 human mammary cancer cells. *Exp. Biol. Med. (Maywood).* **228**, 1078–82 (2003).

45. D. B. Morton, Atypical soluble guanylyl cyclases in Drosophila can function as molecular oxygen sensors. *J. Biol. Chem.* **279**, 50651–3 (2004).

46. K. E. Hillyer, S. Tumanov, S. Villas-Boas, S. K. Davy, Metabolite profiling of symbiont and host during thermal stress and bleaching in a model cnidarian-dinoflagellate symbiosis. *J. Exp. Biol.* **219**, 516–527 (2016).

47. S. R. Dunn, M. C. Thomas, G. W. Nette, S. G. Dove, A Lipidomic Approach to Understanding Free Fatty Acid Lipogenesis Derived from Dissolved Inorganic Carbon within Cnidarian-Dinoflagellate Symbiosis. *PLoS One*. **7**, e46801 (2012).

48. A. B. Imbs, L. P. T. Dang, V. G. Rybin, N. T. Nguyen, L. Q. Pham, Distribution of Very-Long-Chain Fatty Acids between Molecular Species of Different Phospholipid Classes of Two Soft Corals. *Biochem. Anal. Biochem.* **04**, 4–6 (2015).

49. A. B. Imbs, I. M. Yakovleva, T. N. Dautova, L. H. Bui, P. Jones, Diversity of fatty acid composition of symbiotic dinoflagellates in corals: Evidence for the transfer of host PUFAs to the symbionts. *Phytochemistry*. **101**, 76–82 (2014).

50. K. E. Hillyer, D. A. Dias, A. Lutz, U. Roessner, S. K. Davy, Mapping carbon fate during bleaching in a model cnidarian symbiosis: the application of 13 C metabolomics. *New Phytol.* **214**, 1551–1562 (2017).

51. H.-L. Liou *et al.*, NPC2, the Protein Deficient in Niemann-Pick C2 Disease, Consists of Multiple Glycoforms That Bind a Variety of Sterols. *J. Biol. Chem.* **281**, 36710–36723 (2006).

52. Z. Xu, W. Farver, S. Kodukula, J. Storch, Regulation of Sterol Transport between Membranes and NPC2. *Biochemistry*. **47**, 11134–11143 (2008).

53. S. Baumgarten *et al.*, The genome of Aiptasia , a sea anemone model for coral symbiosis. *Proc. Natl. Acad. Sci.* **112**, 11893–11898 (2015).

54. H. M. van Rossum *et al.*, Requirements for carnitine shuttle-mediated translocation of mitochondrial acetyl moieties to the yeast cytosol. *MBio*. **7**, 1–14 (2016).

55. S. Sharma, S. M. Black, Carnitine homeostasis, mitochondrial function and cardiovascular disease. *Drug Discov. Today Dis. Mech.* **6**, e31–e39 (2009).

56. R. K. M. Choy, J. H. Thomas, Fluoxetine-Resistant Mutants in C. elegans Define a Novel Family of Transmembrane Proteins. *Mol. Cell*. **4**, 143–152 (1999).

57. J. L. Watts, J. Browse, Dietary manipulation implicates lipid signaling in the regulation of germ cell maintenance in C. elegans. *Dev. Biol.* **292**, 381–392 (2006).

58. F. Alpy, Give lipids a START: the StAR-related lipid transfer (START) domain in mammals. *J. Cell Sci.* **118**, 2791–2801 (2005).

59. S.-E. Peng *et al.*, Lipid bodies in coral-dinoflagellate endosymbiosis: Proteomic and ultrastructural studies. *Proteomics*. **11**, 3540–3555 (2011).

60. E. I. Christensen, H. Birn, Megalin and cubilin: multifunctional endocytic receptors. *Nat. Rev. Mol. Cell Biol.* **3**, 258–267 (2002).

61. Y. Rong *et al.*, Spinster is required for autophagic lysosome reformation and mTOR reactivation following starvation. *Proc. Natl. Acad. Sci.* **108**, 7826–7831 (2011).

62. A. Bertucci *et al.*, Carbonic anhydrases in anthozoan corals—A review. *Bioorg. Med. Chem.* **21**, 1437–1450 (2013).

63. M. S. Burriesci, T. K. Raab, J. R. Pringle, Evidence that glucose is the major transferred metabolite in dinoflagellate-cnidarian symbiosis. *J. Exp. Biol.* **215**, 3467–3477 (2012).

64. L. F. Whitehead, A. E. Douglas, Metabolite comparisons and the identity of nutrients translocated from symbiotic algae to an animal host. *J. Exp. Biol.* **206**, 3149–57 (2003).

65. M. Hagedorn *et al.*, Trehalose Is a Chemical Attractant in the Establishment of Coral Symbiosis. *PLoS One*. **10**, e0117087 (2015).

66. C. Shinzato *et al.*, Using the Acropora digitifera genome to understand coral responses to environmental change. *Nature*. **476**, 320–323 (2011).

67. L. Feliubadaló *et al.*, Non-type I cystinuria caused by mutations in SLC7A9, encoding a subunit (bo,+AT) of rBAT. *Nat. Genet.* **23**, 52–57 (1999).
